# FastTENET: an accelerated TENET algorithm based on manycore computing in Python

**DOI:** 10.1093/bioinformatics/btae699

**Published:** 2024-11-21

**Authors:** Rakbin Sung, Hyeonkyu Kim, Junil Kim, Daewon Lee

**Affiliations:** Department of Applied Art and Technology, College of Art and Technology, Chung-Ang University, Anseong 17546, Republic of Korea; Department of Bioinformatics, Soongsil University, Seoul 06978, Republic of Korea; Department of Bioinformatics, Soongsil University, Seoul 06978, Republic of Korea; School of Systems Biomedical Science, Soongsil University, Seoul 06978, Republic of Korea; Department of Applied Art and Technology, College of Art and Technology, Chung-Ang University, Anseong 17546, Republic of Korea; School of Art and Technology, College of Art and Technology, Chung-Ang University, Anseong 17546, Republic of Korea

## Abstract

**Summary:**

TENET reconstructs gene regulatory networks from single-cell RNA sequencing (scRNAseq) data using the transfer entropy (TE), and works successfully on a variety of scRNAseq data. However, TENET is limited by its long computation time for large datasets. To address this limitation, we propose FastTENET, an array-computing version of TENET algorithm optimized for acceleration on manycore processors such as GPUs. FastTENET counts the unique patterns of joint events to compute the TE based on array computing. Compared to TENET, FastTENET achieves up to 973× performance improvement.

**Availability and implementation:**

FastTENET is available on GitHub at https://github.com/cxinsys/fasttenet.

## Graphical Abstract

**Graphical Abstract:**
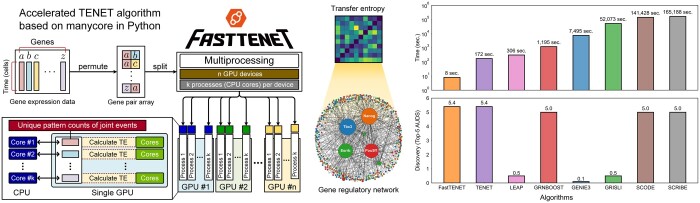

## 1 Introduction

The advent of single-cell RNA sequencing (scRNAseq) has revolutionized our understanding of cellular dynamics. To date, a large amount of scRNAseq data has been accumulated, requiring the development of various algorithms and software for scRNAseq data analysis (Eisenstein 2020, Andrews *et al.* 2021, Granja *et al.* 2021). The reconstruction or inference of a gene regulatory network (GRN) is one of the most important approaches to understand biological mechanisms by analyzing the regulatory relationships between genes at the system level (Pratapa *et al.* 2020, Kim *et al.* 2024).

TENET is a GRN reconstruction tool that employs the transfer entropy (TE) concept in information theory to quantify the strength of the causal relationships between genes from scRNAseq expression data (Kim *et al.* 2021). It has been used to successfully reconstruct GRNs and identify key regulators from various datasets, including mouse embryonic stem cells, cardiomyocyte reprogramming (Kim *et al.* 2021), and mouse embryonic fibroblasts during autophagy (Kim *et al.* 2022).

Furthermore, TENET has broad applicability because it applies the concept of transfer entropy to infer causal relationships between time-dependent variables. TENET has demonstrated superior performance in identifying key regulatory factors in areas such as stem cell differentiation, autophagy, and Parkinson’s disease. It can be used to analyze and elucidate key regulatory factors across various cellular trajectories, including developmental processes and complex disease progression. By identifying critical regulators, TENET can advance the understanding of stem cell engineering and facilitate the discovery of therapeutic targets for complex diseases such as neurodegenerative disorders and cancer.

However, the sequential flow of multiple loops with multiple branches in the original implementation of TE computation creates a performance bottleneck for TENET. This issue becomes more pronounced when analyzing larger datasets or when combining various types of heterogeneous datasets. To improve the performance of TENET, we have developed “FastTENET,” an accelerated implementation of TENET that leverages parallel processing on manycore processors. We have designed array structures specialized for parallel computing and developed parallel algorithms to compute TE based on these structures. In our experiments, FastTENET running on four NVIDIA GeForce RTX 4090 GPU devices has achieved up to a 973× performance improvement over the original TENET running on 32 CPU cores. The performance improvement of FastTENET depends on the characteristics of the given data; specifically, the less diverse the gene expression values within the time series data of a gene pair, the better the performance of FastTENET. FastTENET supports a variety of computing resources, such as CPUs, GPUs, and TPUs (Tensor Processing Units), as its array computing functionality is powered by various acceleration frameworks including NumPy (Harris *et al.* 2020), CuPy (Okuta *et al.* 2017), JAX (Bradbury *et al.* 2018), TensorFlow (Abadi *et al.* 2015), PyTorch (Paszke *et al.* 2019), and PyTorch Lightning (Falcon *et al.* 2019).

## 2 Materials and methods

### 2.1 Basic concept

TENET quantifies the amount of putative causal relationships between genes from scRNAseq data by calculating bidirectional pairwise TEs for selected genes. The TE from *Y* to *X* is defined by Equation (1).

(1) $$\begin{array}{c} TE_{Y\to X}=H(X_{t+1}|X_{t})-H(X_{t+1}|X_{t},Y_{t})\\ =\sum p(X_{t+1},X_{t},Y_{t})\log\frac{p(X_{t+1},X_{t},Y_{t})p(X_{t})}{p(X_{t+1},X_{t})p(X_{t},Y_{t})}, \end{array}$$

where *H*(*X*) is Shannon’s entropy of *X* and *t* is the time. The TE calculates the amount by which the uncertainty in $X_{t+1}$ is reduced by the information in *Y_t_* (Supplementary Fig. S1). According to the definition of Shannon’s entropy, TE can be expressed as a sum of joint probabilities. To obtain the joint probabilities, TENET counts the occurrence of joint events, $\left(X_{t+1},X_{t},Y_{t}),\;(X_{t+1},X_{t}\right)$, (*X_t_*, *Y_t_*), and $\left(X_{t}\right)$, in the time series data of a gene pair (*X*, *Y*). Given that the gene expression values in scRNAseq data are typically continuous, it is necessary to discretize the values (i.e. a bin index must be assigned to each value) to count the joint events and thereby approximate the TE (Supplementary Figs S2–S5). TENET performs ${}_{n}P_{2}$ TE computations, considering all possible bidirectional relationships between all *n* genes.

### 2.2 Data structures for array computing

**Figure 1.**
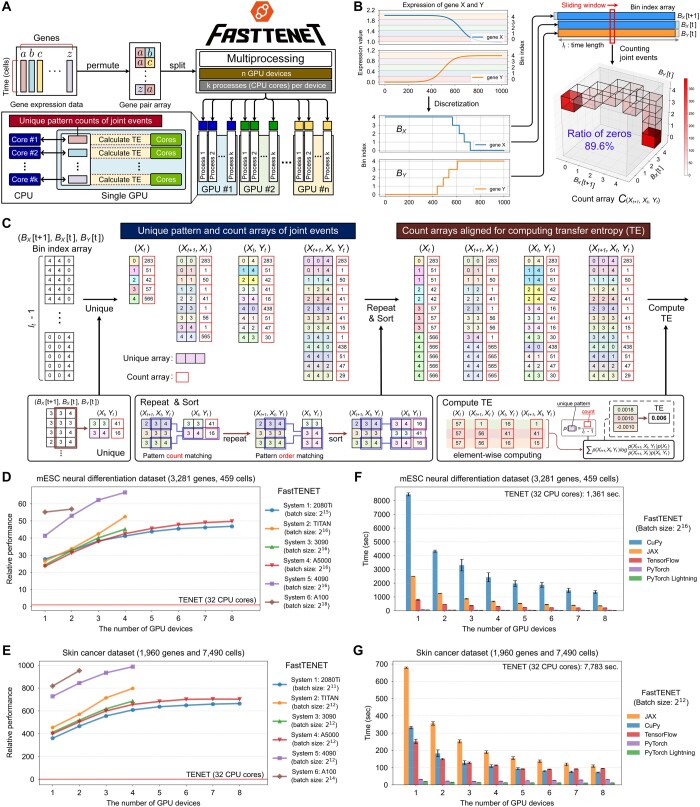
Overview of FastTENET and performance comparison. (A) Workflow of parallel processing. (B) A toy example of the count array $C_{\left(X_{t+1},X_{t},Y_{t}\right)}$ in Equation (2), illustrating extreme sparsity. (C) An example of creating count arrays specialized for parallel computing in FastTENET. (D and E) Relative performances of FastTENET on the (D) mESC neural differentiation dataset and (E) skin cancer dataset across various computing systems. All experiments of original TENET were conducted with Intel Xeon Silver 4214R 32 CPU cores. (F and G) Execution times of FastTENET across various manycore acceleration frameworks on the (F) mESC neural differentiation dataset and (G) skin cancer dataset, utilizing NVIDIA A5000 GPUs in System 4. Refer to Supplementary Table S1 for detailed configurations of the computing systems.

To accelerate the computation of TENET, we have employed an array computing approach with parallel processing on manycore processors (Fig. 1A). The first step in array computing is the creation of array data structures that can be distributed evenly to multiple workers for parallel processing. As the TE computation requires counting the four types of joint events to calculate the joint probabilities in Equation (1), it is necessary to design the count array for array computing. The most intuitive way to design the count array is to interpret the discretized values or bin indices of the gene expression at time *t* as the indices of the count array (Fig. 1B and Supplementary Figs S6 and S7). For example, if we count the joint event $\left(X_{t+1},X_{t},Y_{t}\right)$ for a gene pair (*X*, *Y*), then the count array of joint event $\left(X_{t+1},X_{t},Y_{t}\right)$ can be represented as $C_{\left(X_{t+1},X_{t},Y_{t}\right)}$ in Equation (2).

(2) $$\begin{array}{c} C_{\left(X_{t+1},X_{t},Y_{t}\right)}[i,j,k]=\sum_{t=1}^{l_{t}-1}f(B_{X}[t+1],B_{X}[t],B_{Y}[t]),\\ f(x,y,z)=\{\begin{array}{cc} 1,\; & \text{if}\;x=i,\;y=j,\;z=k,\\ 0,\; & \text{otherwise}, \end{array} \end{array}$$

where *l_t_* is the length of time and *B_X_* is the bin index of the expression values of gene *X*. The size of the count array *C* is determined by the size of the largest bin index array *B* in Equation (2). However, this approach has a critical limitation. The count array exhibits extreme sparsity due to the wide range of expression values present in the time series data for each gene, as well as the low frequency of joint event patterns.

Figure 1B shows a toy example of the count array with extreme sparsity. The expression of gene *X* and gene *Y* is synthetic data. These values are discretized into five categories and represented as discrete patterns. The occurrences of four joint events are counted from the bin index array. In this example, the count array $C_{\left(X_{t+1},X_{t},Y_{t}\right)}$ has extreme sparsity: the proportion of zero values is 89.6%. This sparsity frequently leads to out-of-memory errors, thereby constraining the batch size applicable for parallel processing.

To overcome this limitation by ensuring that memory space is not wasted, we have developed array data structures that consider only the patterns of joint events present in the discretized time series data, rather than all possible patterns (Fig. 1C). FastTENET identifies and counts the four joint event types from the discretized time series data of all gene pairs to create the four count arrays of joint events. A crucial step in creating the four count arrays involves repeating and sorting the patterns in each count array, thereby ensuring the alignment of pattern positions across the count arrays for array computing (Fig. 1C and Supplementary Figs S8–S17).

### 2.3 Parallel processing on manycore processors

FastTENET supports parallel processing on manycore processors to compute the TE. Users can select multiple CPU cores for multiprocessing and manycore processors for accelerating the array computing. FastTENET divides scRNAseq data into multiple batches according to the available computing resources. In other words, it spawns multiple processes, and each process is assigned to a specific manycore device to compute the TE of a single batch. Users need to set the available manycore devices, number of processes per device, and batch size per device for parallel processing. The results of each batch are combined into a single array through the shared memory among the multiple processes (Fig. 1A). The mathematical operations of array computing can be significantly accelerated by manycore processors, such as GPUs or TPUs. The array computing of FastTENET is supported by various acceleration frameworks such as CuPy, JAX, TensorFlow, PyTorch, and PyTorch Lightning.

### 2.4 Experimental setup

We conducted experiments with two scRNAseq datasets (Supplementary Section S4.2) to evaluate the performance of FastTENET compared to TENET. The first dataset is the mESC neural differentiation dataset; it was utilized for evaluating TENET (Setty *et al.* 2016, Tuck *et al.* 2018). In our experiment, we used the mESC dataset consisting of 3281 highly variable genes and 459 cells. The second dataset is the skin cancer dataset, which is constructed by integrating multiple datasets obtained from the GEO database (Ji *et al.* 2020, Gaydosik *et al.* 2020, Kfoury *et al.* 2021). In our experiment, the skin cancer dataset consists of 1960 highly variable genes and 7490 cells.

The performance comparison was conducted on eight different computing systems (Supplementary Table S1). The baseline for the performance comparison was defined as the execution time of TENET on System 4 with 32 CPU cores. The execution times of FastTENET were measured by varying the number of GPU devices. The maximum number of GPU devices varies from system to system due to heterogeneous hardware configurations. The batch size of each process was determined by the given dataset to maximize the GPU memory usage. The experiments were repeated 10 times for both datasets. The relative performance of FastTENET was obtained by dividing its execution time by that of TENET. In addition, we have also analyzed the performance of the manycore acceleration frameworks of FastTENET on System 4. Each experiment was repeated 10 times on both datasets, with incremental scaling up to eight GPU devices.

## 3 Results

### 3.1 Performance improvement

The maximum performance improvement of FastTENET on the mESC dataset was 67× when utilizing the maximum capacity of the GPU resources in System 5 (Fig. 1D). On System 5, the average computation time of TENET on the mESC dataset was 1361 s (22.68 min), whereas FastTENET required only 20 s (0.34 min) with the maximum hardware utilization (Supplementary Fig. S24A). For the skin cancer dataset, FastTENET achieved a significant performance improvement of up to 973× (Fig. 1E). The performance of FastTENET was further improved on both datasets by utilizing more GPU devices (Fig. 1D and E). On the skin cancer dataset, the average execution time of TENET was 7783 s (129.71 min). FastTENET, on the other hand, could reduce the execution time to 7.8 s (0.13 min), which was about 973 times faster than TENET (Supplementary Fig. S24B). Interestingly, the actual execution time for the skin cancer dataset is much lower than that for the mESC neural differentiation dataset, even though the size of the skin cancer dataset is approximately 9.7 times larger than that of the mESC dataset. This is because the number of unique patterns associated with the joint events, rather than the data size, critically affects the execution time of FastTENET (Supplementary Figs S23 and S24, Supplementary Tables S3 and S4). The overall experimental results for the mESC and skin cancer datasets suggest that FastTENET is considerably faster than TENET. However, it is noteworthy that the extent of performance improvement may vary depending on the scRNAseq data under consideration.

### 3.2 Manycore acceleration framework

The computation time of FastTENET with PyTorch Lightning was minimal compared to the other frameworks (Fig. 1F and G). In contrast, the average computation time of FastTENET with CuPy on the mESC dataset was 8520 s (142 min), demonstrating the slowest performance when utilizing a single A5000 GPU (Fig. 1F). On the skin cancer dataset, JAX was the slowest framework when utilizing a single A5000 GPU, with the average computation time of 1308 s (21.8 min) (Fig. 1G). The variation in performance ranking between CuPy and JAX across datasets is attributed to differences in their implementations of “unique” and “repeat” functions (Supplementary Fig. S18).

### 3.3 In-depth performance analysis

Further experiments were conducted to analyze the impact of parameter conditions and data size on the execution times of FastTENET (Supplementary Figs S19–S22). The results indicate that utilizing the maximum available resources is recommended for FastTENET. However, optimal parameter conditions should be heuristically decided due to the impact of the data characteristics (e.g. the number of unique patterns) on the performance of FastTENET. The TE approximations of FastTENET and TENET were compared to identify key hub regulators by varying the discretization methods used in FastTENET (Supplementary Figs S26–S28). Moreover, we evaluated the performance of FastTENET in terms of execution time and its ability to identify key hub regulators, comparing it with other GRN reconstruction algorithms. Additionally, we analyzed the impact of discretizing and smoothing functions on the discovery score achieved by FastTENET (Supplementary Figs S25, S29 and S30).

## 4 Conclusion

FastTENET is an accelerated implementation of TENET based on manycore parallel processing. To accelerate the TENET algorithm, we have developed the array data structures specialized for array computing on manycore processors with multiprocessing. FastTENET demonstrates scalable performance improvement in inferring GRNs from large-scale scRNAseq datasets by leveraging manycore devices. FastTENET incorporates an abstraction layer that unifies various manycore acceleration frameworks into a single API. Consequently, the performance of FastTENET is expected to improve as the acceleration frameworks evolve alongside future manycore devices. We expect that the advancement of manycore devices will lead to improvements in the computation speed of FastTENET without requiring any modifications.

## Supplementary Material

btae699_Supplementary_Data

## Data Availability

FastTENET with test dataset is available online for public use at https://github.com/cxinsys/fasttenet.

## References

[btae699-B1] Abadi M , Agarwal A, Barham P et al TensorFlow. GitHub repository. 2015. https://github.com/tensorflow/tensorflow (30 October 2024, date last accessed).

[btae699-B2] Andrews TS , Kiselev VY, McCarthy D et al Tutorial: guidelines for the computational analysis of single-cell RNA sequencing data. Nat Protoc 2021;16:1–9. 33288955 10.1038/s41596-020-00409-w

[btae699-B3] Bradbury J, Frostig R, Hawkins P et al JAX. GitHub repository. 2018. https://github.com/jax-ml/jax (30 October 2024, date last accessed).

[btae699-B4] Eisenstein M. Single-cell RNA-seq analysis software providers scramble to offer solutions. Nat Biotechnol 2020;38:254–7. 10.1038/s41587-020-0449-8 32152578

[btae699-B5] Falcon W, Borovec J, Schock J et al PyTorch Lightning. GitHub repository. 2019. https://github.com/Lightning-AI/pytorch-lightning (30 October 2024, date last accessed).

[btae699-B6] Gaydosik AM , Queen DS, Trager MH et al Genome-wide transcriptome analysis of the STAT6-regulated genes in advanced-stage cutaneous T-cell lymphoma. Blood 2020;136:1748–59. 32438399 10.1182/blood.2019004725 PMC7544545

[btae699-B7] Granja JM , Corces MR, Pierce SE et al ArchR is a scalable software package for integrative single-cell chromatin accessibility analysis. Nat Genet 2021;53:403–11. 33633365 10.1038/s41588-021-00790-6 PMC8012210

[btae699-B8] Harris CR , Millman KJ, van der Walt SJ et al Array programming with NumPy. Nature 2020;585:357–62. 32939066 10.1038/s41586-020-2649-2 PMC7759461

[btae699-B9] Ji AL , Rubin AJ, Thrane K et al Multimodal analysis of composition and spatial architecture in human squamous cell carcinoma. Cell 2020;182:497–514.e22. 32579974 10.1016/j.cell.2020.05.039 PMC7391009

[btae699-B10] Kfoury Y , Baryawno N, Severe N et al; as part of the Boston Bone Metastases Consortium. Human prostate cancer bone metastases have an actionable immunosuppressive microenvironment. Cancer Cell 2021;39:1464–78.e8. 34719426 10.1016/j.ccell.2021.09.005 PMC8578470

[btae699-B11] Kim D , Kim J, Yu YS et al Systemic approaches using single cell transcriptome reveal that C/EBP*γ* regulates autophagy under amino acid starved condition. Nucleic Acids Res 2022;50:7298–309. 35801910 10.1093/nar/gkac593 PMC9303372

[btae699-B12] Kim H , Choi H, Lee D et al A review on gene regulatory network reconstruction algorithms based on single cell RNA sequencing. Genes Genomics 2024;46:1–11. 38032470 10.1007/s13258-023-01473-8

[btae699-B13] Kim J , T Jakobsen S, Natarajan KN et al TENET: gene network reconstruction using transfer entropy reveals key regulatory factors from single cell transcriptomic data. Nucleic Acids Res 2021;49:e1. 33170214 10.1093/nar/gkaa1014 PMC7797076

[btae699-B14] Okuta R, Unno Y, Nishino D et al CuPy: a NumPy-compatible library for NVIDIA GPU calculations. In: *Proceedings of Workshop on Machine Learning Systems (LearningSys) in the 31st Annual Conference on Neural Information Processing Systems,* Long Beach, CA, USA, Vol. 151(7). 2017.

[btae699-B15] Paszke A, Gross S, Massa F et al PyTorch: an imperative style, high-performance deep learning library. In: *Proceedings of the 33rd International Conference on Neural Information Processing Systems*. Red Hook, NY, USA, Article 721, pp. 8026–8037. 2019.

[btae699-B16] Pratapa A , Jalihal AP, Law JN et al Benchmarking algorithms for gene regulatory network inference from single-cell transcriptomic data. Nat Methods 2020;17:147–54. 31907445 10.1038/s41592-019-0690-6 PMC7098173

[btae699-B17] Setty M , Tadmor MD, Reich-Zeliger S et al Wishbone identifies bifurcating developmental trajectories from single-cell data. Nat Biotechnol 2016;34:637–45. 27136076 10.1038/nbt.3569 PMC4900897

[btae699-B18] Tuck AC , Natarajan KN, Rice GM et al Distinctive features of lincRNA gene expression suggest widespread RNA-independent functions. Life Sci Alliance 2018;1:e201800124. 30456373 10.26508/lsa.201800124 PMC6238598

