# Supplementary material for: FastTENET: an accelerated TENET algorithm based on manycore computing in Python
Source: Bioinformatics. 2024 Nov 21;40(12):btae699. doi: 10.1093/bioinformatics/btae699 (PMC11645435; doi:10.1093/bioinformatics/btae699)
Supplement: btae699_Supplementary_Data [file btae699_supplementary_data.pdf]

Supplementary Data

FastTENET: an accelerated TENET algorithm based on manycore computing in Python

Rakbin Sung[1], Hyeokyu Kim[2], Junil Kim[2,3], and Daewon Lee[1,4]

[1] *Department of Applied Art and Technology, Chung-Ang University, Anseong 17546, Republic of Korea*
[2] *School of Systems Biomedical Science, Soongsil University, Seoul 06978, Republic of Korea*
[3] *Department of Bioinformatics, Soongsil University, Seoul 06978, Republic of Korea*
[4] *School of Art and Technology, Chung-Ang University, Anseong 17546, Republic of Korea*

Contents

1 Introduction **2**

2 Basic concept of TENET **2**
2.1 Transfer entropy . . . 3
2.2 Computation of transfer entropy . . . 3
2.2.1 Rearrangement of expression data . . . 4
2.2.2 Discretization . . . 4
2.2.3 Computation of transfer entropy . . . 5
2.2.4 Interpretation of computation results . . . 6

3 FastTENET **7**
3.1 Computational limitations in TENET . . . 7
3.2 Representation of discretized values as array indices . . . 8
3.3 Computation procedure . . . 10
3.3.1 Integration of joint event patterns . . . 10
3.3.2 Identification of unique patterns . . . 11
3.3.3 Rearrangement of count arrays . . . 13
3.3.4 Computation of transfer entropy . . . 17
3.4 Parallel processing on manycore processors . . . 18
3.5 Utilization . . . 19
3.5.1 Initialization . . . 19
3.5.2 Execution . . . 19

4 Performance analysis 21
4.1 Computing systems 21
4.2 Dataset overview 22
4.3 Performance metrics 23
4.4 Analysis of computational speed 24
4.4.1 Analysis of acceleration framework 24
4.4.2 Impact of the number of processors 27
4.4.3 Impact of batch size 28
4.4.4 Impact of data size 28
4.4.5 Impact of expression patterns 31
4.4.6 Evaluation of GRN algorithms for computational speed 34
4.5 Approximation of transfer entropy 35
4.5.1 Comparison of approximation results 35
4.5.2 Comparison of smoothing and discretizing functions 36
4.5.3 Evaluation of GRN algorithms for transcription factor discovery 40

1 Introduction

A GitHub repository has been made available to support the open-source development of the FastTENET framework. You can find the implementation in Python and helpful tutorials to get started with FastTENET in the repository.

- Home: https://github.com/cxinsys/fasttenet
- Tutorials: https://github.com/cxinsys/fasttenet/tree/main/tutorials

In this supplementary data, we have introduced the FastTENET framework, a manycore accelerated computing implementation of TENET using Python.

The FastTENET framework has the following main features.

1. Design specialized data structures for array computing
2. Parallel processing on manycore processors such as GPUs or TPUs

2 Basic concept of TENET

TENET is a software to reconstruct a gene regulatory network (GRN) from scRNAseq expression data. To reconstruct the GRN, TENET quantifies the strength of the causal

relationship between genes by computing transfer entropy (TE). This section presents an overview of the transfer entropy computation algorithm of TENET.

2.1 Transfer entropy

TE is a nonparametric statistical approach for measuring the directed transfer of information between two random processes. The TE from process Y to process X is defined as the reduction in the uncertainty of the value of X at a future point in time resulting from the addition of information about the value of Y at a previous point in time to the previous value of X. The formula for TE from process Y to process X can be expressed as Equation 1.

$$TE_{Y \to X} = H(X_{t+1}|X_t) - H(X_{t+1}|X_t, Y_t) \tag{1}$$

where $H(X_{t+1}|X_t)$ is the conditional entropy of the probability of event $X(t+1)$ occurring given that event $X(t)$ occurred. The TE can be calculated by taking the difference between the two conditional entropies, $H(X_{t+1}|X_t)$ and $H(X_{t+1}|X_t, Y_t)$. For example, we can consider the gene expression data from two genes, X and Y. If Y has a strong influence on X, the value of the conditional entropy $H(X_{t+1}|X_t, Y_t)$ would decrease as the uncertainty is reduced due to the additional information provided by Y. Consequently, the value of the TE would increase. Conversely, if Y has a weak influence on X, the difference between the two conditional entropies would be small, resulting in the value of the TE. Figure S1 illustrates cases of both strong and weak influences of Y on X.

2.2 Computation of transfer entropy

TENET computes the TE between all genes and reconstructs a gene network based on the TE values. This section describes the TE computation process of TENET.

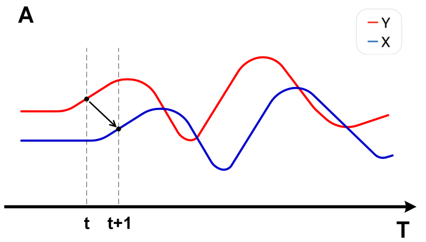

Figure S1. Examples of gene expression data illustrating a **(A)** strong or **(B)** weak influence of gene Y on gene X.

2.2.1 Rearrangement of expression data

To calculate the TE, expression data, a time series representation of the expression of a gene, is prepared. Then, this data is preprocessed through some steps, including by selecting and sorting according to the time point (Fig. S2).

expression data

	Gene A	Gene B
chip_063_cell_10	33	2518
chip_063_cell_11	0	2460
chip_063_cell_12	10	2383
chip_063_cell_13	1	2704
chip_063_cell_14	1	2591
chip_063_cell_15	4	3716
chip_063_cell_16	1	1809
chip_063_cell_17	15	1419
chip_063_cell_18	4	1478
chip_063_cell_19	0	3273
chip_063_cell_20	0	1980
chip_063_cell_21	1	2423
chip_063_cell_22	1	2152
chip_063_cell_23	4	1899

+

time point selection data

branch
0
1
1
1
1
1
0
0
0
1
1
1
0
1

+

time point sorted data

trajectory
0.011202
0.024460
0.013835
0.072500
0.004479
0.019142
0.035321
0.013806
0.029426
0.019236
0.069377
0.089212
0.021779
0.054252

→

rearranged data

	Gene A	Gene B
chip_063_cell_14	1	2591
chip_063_cell_12	10	2383
chip_063_cell_15	4	3716
chip_063_cell_19	0	3273
chip_063_cell_11	0	2460
chip_063_cell_23	4	1899
chip_063_cell_20	0	1980
chip_063_cell_13	1	2704
chip_063_cell_21	1	2423

Figure S2. Example of rearranging data.

2.2.2 Discretization

Prior to computing TE from expression data, TENET applies discretization, a preprocessing step known as binning. Binning is a type of discretization that divides continuous values into discrete bins, thereby converting the values into integers. The following equation describes the discretization:

$$X_b = \lfloor \frac{X - min(X)}{kw} \rfloor, \quad kw = \kappa * std(X), \tag{2}$$

where X, X_b, kw, and κ are gene expression data, binned data, kernel width, and kernel coefficient, respectively. After binning, the data will have more discrete values than before. Figure S3 shows an example of the data before and after binning.

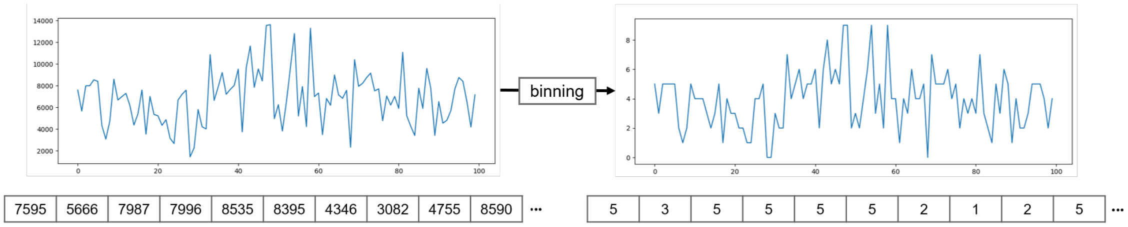

Figure S3. Example of data changes before and after binning.

2.2.3 Computation of transfer entropy

To understand how to compute the TE in TENET, we consider the TE equation. By the definition of conditional entropy, the TE can be expressed as a sum of joint probabilities (Equation 3).

$$\begin{aligned} TE_{Y \to X} &= H(X_{t+1}|X_t) - H(X_{t+1}|X_t, Y_t) \\ &= \sum p(X_{t+1}, X_t, Y_t) \log \frac{p(X_{t+1}, X_t, Y_t) p(X_t)}{p(X_{t+1}, X_t) p(X_t, Y_t)} \end{aligned} \tag{3}$$

As shown in Equation 3, to compute the TE from Y to X, we need information about four joint probabilities: $p(X_{t+1}, X_t, Y_t)$, $p(X_{t+1}, X_t)$, $p(X_t, Y_t)$, and $p(X_t)$. These joint probabilities can be respectively calculated by dividing the frequency of joint events, (X_{t+1}, X_t, Y_t), (X_{t+1}, X_t), (X_t, Y_t), and (X_t), by the time t.

If we have expression data observed at 12 time points, we can count the joint event (X_{t+1}, X_t, Y_t) as shown in Figure S4. Further, because 12 time points exist, the

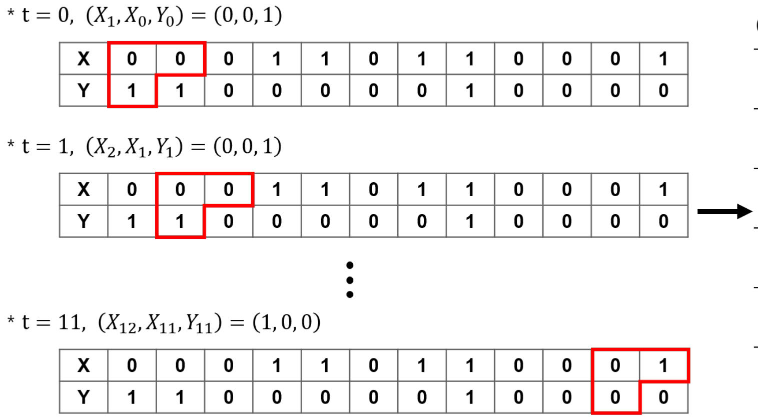

(X_{t+1}, X_t, Y_t)	Count	$p(X_{t+1}, X_t, Y_t)$
(0, 0, 0)	2	$\frac{2}{11}$
(0, 0, 1)	2	$\frac{2}{11}$
(0, 1, 0)	1	$\frac{1}{11}$
(0, 1, 1)	1	$\frac{1}{11}$
(1, 0, 0)	3	$\frac{3}{11}$
(1, 1, 0)	2	$\frac{2}{11}$

Figure S4. Example of calculating probability for joint events in TENET algorithm.

total number of events that have occurred is 11. Therefore, we can easily obtain the probability of the joint event from the data; for example, $p(0, 0, 0) = \frac{2}{11}$. Similarly, we can obtain the probability of other joint events, including $p(X_{t+1}, X_t)$, $p(X_t, Y_t)$, $p(X_t)$ from the data.

2.2.4 Interpretation of computation results

$TE_{B \to C}$

source

target

	A	B	C	D	E	F	G	H	I	J
A	0	0.290885	0.135615	0.273601	0.261619	0.253119	0.203788	0.331363	0.289781	0.28243
B	0.392627	0	0.260536	0.516161	0.598121	0.469311	0.41415	0.642225	0.511272	0.457406
C	0.151807	0.209684	0	0.210652	0.194273	0.226172	0.174159	0.188985	0.249431	0.222713
D	0.357282	0.477406	0.244749	0	0.439551	0.468899	0.381829	0.562088	0.449024	0.449687
E	0.308202	0.501803	0.278862	0.402002	0	0.427866	0.289438	0.504811	0.439091	0.413023
F	0.369399	0.471613	0.238156	0.444637	0.486199	0	0.37914	0.593352	0.409917	0.392599
G	0.219433	0.268068	0.190455	0.29503	0.260383	0.276596	0	0.322946	0.292247	0.252262
H	0.448317	0.674412	0.290522	0.605538	0.57539	0.605783	0.433281	0	0.639565	0.606809
I	0.33306	0.520994	0.268104	0.452111	0.542908	0.427644	0.403597	0.661831	0	0.351188
J	0.373866	0.477331	0.259857	0.519558	0.495653	0.427245	0.360569	0.645896	0.33858	0

Figure S5. Example of result matrix of TE in TENET.

Finally, the result matrix is constructed as an array containing the TE values for all gene pairs. The result matrix is a square matrix in which each column represents the source gene and each row represents the target gene for each gene pair. For example, $TE_{B \to C}$ represents the transfer entropy from gene B (source) to gene C (target) (Fig. S5).

3 FastTENET

3.1 Computational limitations in TENET

TENET sequentially computes the TE between all gene pairs. The total number of computation loops is ${}_nP_2 = n \times (n-1)$, where n is the number of genes. This is because the TE is directional, whereby $TE_{Y \to X}$ and $TE_{X \to Y}$ are different. The sequential computation method of TENET is inefficient and takes a very long time, as the number of gene pairs increases proportionally to the square of the number of genes. For instance, for data with 10,000 genes, 10,000 $\times$ 9,999 = 99,990,000 TEs need to be calculated.

TENET also computes the TE by multiple loops along the time axis. As shown in Figure S4, TENET counts the occurrences of joint events by observing each time point. Unlike in the previous case, the number of loops does not scale in proportion to the square of the number of genes. However, the computation still slows down as the number of loops increases.

TENET employs multiple branches (i.e., multiple if-else blocks) to count the occurrences of unique joint events. TENET checks whether a pattern of the joint event has been observed in previous time points. If a specific pattern of the joint event has never been observed previously in the time series data, TENET creates an array to count that joint event pattern. If it occurred in the previous time points, TENET finds the array that counts the occurrence of that joint event pattern and adds the occurrence. These branches occur for each joint event (X_{t+1}, X_t, Y_t), (X_{t+1}, X_t), (X_t, Y_t), and (X_t) at a given time point.

To address these limitations, FastTENET is developed with a specialized array structure to accelerate the computational speed of TENET. The data structures of FastTENET, unlike that of TENET, are designed to compute the TEs for all time points and multiple gene pairs based on array computing.

3.2 Representation of discretized values as array indices

FastTENET is developed to accelerate the computation of TE by designing array structures that count the occurrences of joint event patterns for gene pairs based on array computing. The most intuitive approach to design the count arrays is to interpret the discretized values or bin indices of the gene expression at time t as the index of the count array. After creating four count arrays corresponding to the four types of joint events, (X_{t+1}, X_t, Y_t), (X_{t+1}, X_t), (X_t, Y_t), and (X_t), we can treat the discretized values of a joint event at a particular time as the indices of the count arrays. In other words, we can count the joint event patterns by interpreting the discretized values as the indices of count arrays (see also Equation (2) in the main manuscript).

A

X	2	1	3	4	5	5	5	3	3	1	1	1
Y	1	1	0	0	2	1	0	1	0	0	0	0

B

X(t+1)	1	3	4	5	5	5	3	3	1	1	1
X(t)	2	1	3	4	5	5	5	3	3	1	1
Y(t)	1	1	0	0	2	1	0	1	0	0	0

(1,2,1) += 1 (1,1,0) += 1

C

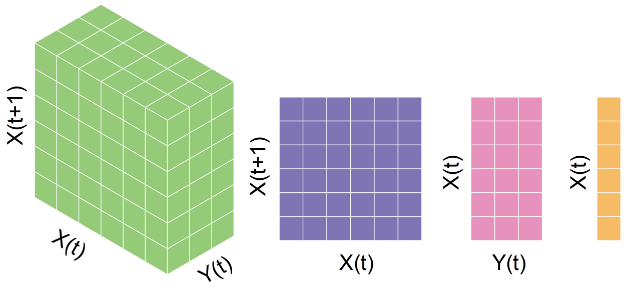

Figure S6. Example of intuitive approach to create count array. **(A)** Example of data with genes X and Y, **(B)** example of reorganizing the data for a (X_{t+1}, X_t, Y_t) joint event array and **(C)** example of generating a multi-dimensional count array for each joint event.

Figure S6A shows the expression data of gene X with values between 0 and 5 and that of gene Y with values between 0 and 2. We can create an array for the (X_{t+1}, X_t, Y_t) joint event by reorganizing the data (Fig. S6B). By observing the pattern of each joint

event at time t and treating it as an index, we can create a count array for the joint event, as shown in Figure S6C.

However, this approach has a couple of issues. First, different genes have different ranges of expression values after discretization, and this changes the shape of the count array. Figure S7A shows three genes X, Y, and Z with different ranges of expression values. An examination of the shape of the joint event count array for these genes clearly reveals that they are different (Fig. S7B). We can resize the other smaller arrays to match the shape of the largest array and then combine them into a single array; however, this approach would result in memory waste.

A

Gene	X	Y	Z
Range of values	$0 \leq X_t \leq 5$	$0 \leq Y_t \leq 2$	$0 \leq Z_t \leq 4$

B

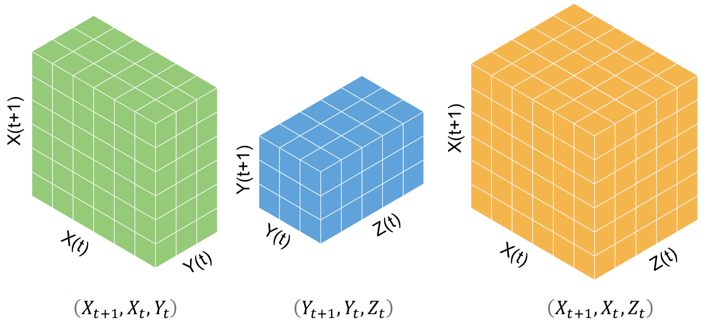

Figure S7. Example of joint event count arrays with different sizes.

The second issue is that the number of non-occurring events is significantly larger than that of occurring events. For instance, as shown in Figure S6, the number of (X_{t+1}, X_t, Y_t) joint event that can occur from the expression data of gene X, where $0 \leq X < 6$ and gene Y, where $0 \leq Y < 3$, is 108 (6×6×3). However, only 10 distinct joint event patterns are actually observed in the data (Fig. S6B). This results in a sparse matrix for the pattern count array, with many elements having zero values.

The same applies to the example in the real dataset. The scRNAseq dataset is acquired from mESC with the 'Rn7sk' and 'Eef1a1' genes treated as X and Y genes, respectively; the array shape of the (X_{t+1}, X_t, Y_t) pattern is (10, 10, 6), and the total size

of the array is 600 (Tuck *et al.*, 2018). The ratio of zero to non-zero elements is 554:46, that is, approximately 92% to 8%. In this data, approximately 92% of the patterns never appear, implying that they have no impact on the TE. Therefore, creating the count array with the index of the array representing the values of joint events is highly inefficient in terms of memory management.

3.3 Computation procedure

To implement the array-based TE computation algorithm on manycore processors, Fast-TENET has designed array structures specialized for parallel computing. This section presents an overview of this array structure (Fig. S8).

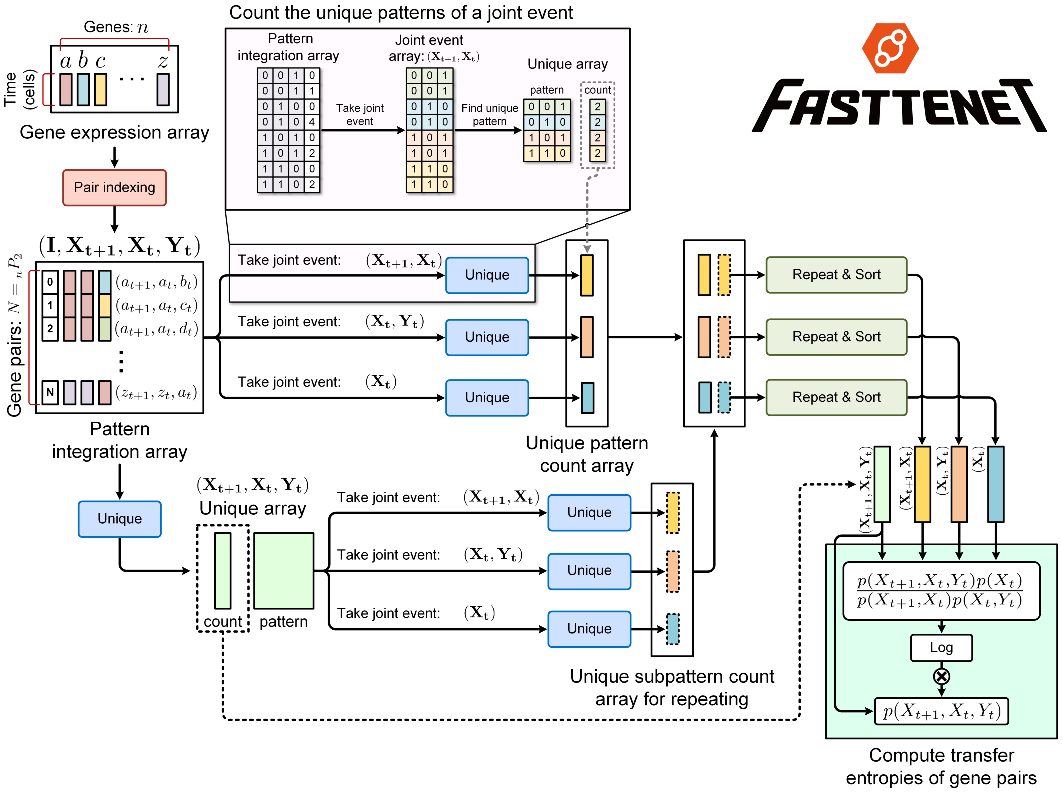

Figure S8. Overview of TE computation algorithm.

3.3.1 Integration of joint event patterns

FastTENET rearranges the data to count the patterns of joint events. The pattern integration array is designed to count the occurrences of each joint event for multiple

gene pairs at once. It is constructed by concatenating the joint event array from the (X_{t+1}, X_t, Y_t) pattern with the gene pair index which is an index of the gene pair permutation.

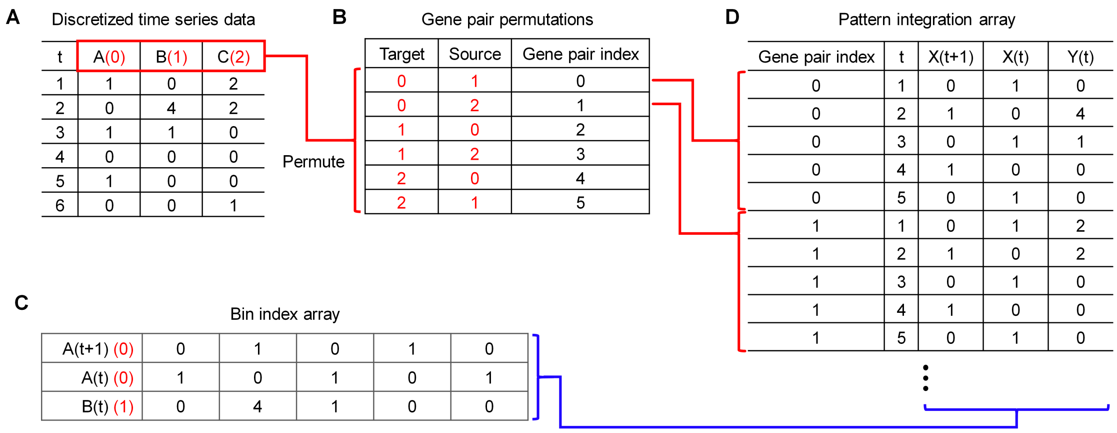

Figure S9. Example of creating the pattern integration array.

Figure S9 shows how we rearrange data with the example of six observations and three genes to create a pattern integration array. Because three genes exist, six permutations of gene pairs are possible. We created the gene pair index for gene pair permutations, as shown in Figure S9. Furthermore, we created bin index arrays based on the gene pair permutations. Finally, to create the pattern integration array, we duplicated the gene pair index for each observation time $t-1$ (in the above example, $t_{total} = 5$) and placed it in the first column to match the shape of the bin index array. As shown in Figure S9, the pattern integration array is two-dimensional: (number of gene pair permutations $\times$ t_{total}, 4).

3.3.2 Identification of unique patterns

The NumPy library provides `unique` function that returns only the unique value patterns in a given multidimensional array. The function allows for the specification of the axis to identify unique value patterns along that axis. Furthermore, setting the `return_counts` parameter to `True` is necessary to obtain an array that contains the counts of each unique value pattern within the array. `numpy.unique` returns the unique values identified along the row axis (`axis=0`) in a two-dimensional array and the number

of occurrences of each unique value (Code 1).

Code 1. Example code of the `unique` function in NumPy

```
import numpy as np

arr = np.array([[1, 2],
                [3, 4],
                [1, 2],
                [5, 6],
                [3, 4]])
unique_arr, counts = np.unique(arr, axis=0, return_counts=True)

print(unique_arr)
# Output:
# array([[1, 2],
#        [3, 4],
#        [5, 6]])

print(counts)
# Output:
# array([2, 2, 1], dtype=int64)
```

Code 1 shows the unique pattern array and its corresponding count array. `numpy.unique` function allows to count only unique patterns for each gene pair. Previously, we added the gene pair index to the first column of the bin index array, which allows the unique function to count unique patterns separately for each gene pair (Fig. S9). Figure S10 illustrates the process of utilizing the unique function to return the count array from the (X_{t+1}, X_t, Y_t) joint event pattern in the pattern integration array.

As shown in Figure S10, the unique pattern and count array for the (X_{t+1}, X_t, Y_t) joint event pattern can be derived directly by utilizing the unique function on the pattern integration array. However, other unique patterns of joint events, such as (X_{t+1}, X_t), (X_t, Y_t), and (X_t), cannot be derived directly from pattern integration array. Selecting a specific array for each joint event from the pattern integration array is

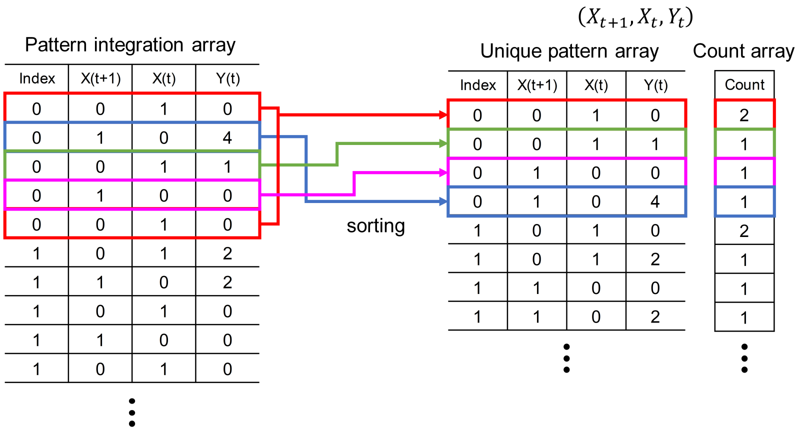

Figure S10. Example of utilizing unique function for (X_{t+1}, X_t, Y_t) joint event.

necessary. Subsequently, the unique pattern and count array can be derived from the specific arrays of joint events (X_{t+1}, X_t), (X_t, Y_t), and (X_t) (Fig. S11).

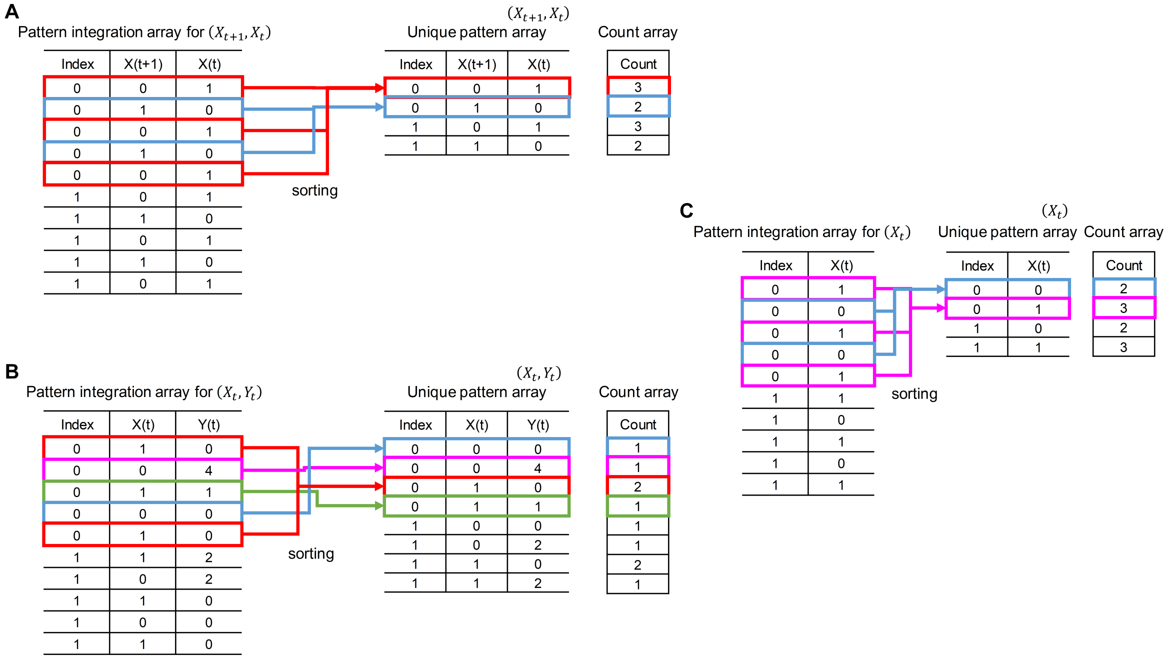

Figure S11. Examples of creating unique pattern and count arrays for joint events **(A)** (X_{t+1}, X_t), **(B)** (X_t, Y_t), and **(C)** (X_t).

3.3.3 Rearrangement of count arrays

The unique pattern count arrays of all joint event counts have been obtained by utilizing the unique function, but we need to rearrange each array to compute the TE.

To compute the TE, the count arrays must have the same shape and alignment. For

instance, the computation of the TE for the pattern $(X_{t+1}, X_t, Y_t) = (0, 1, 2)$, $(1, 0, 2)$, $(1, 1, 1)$, $(1, 1, 2)$, can be expressed as Equation 4.

$$
\begin{aligned}
TE_{Y \to X} &= \sum p(X_{t+1}, X_t, Y_t) \log \frac{p(X_{t+1}, X_t, Y_t) p(X_t)}{p(X_{t+1}, X_t) p(X_t, Y_t)} \\
&= p(0,1,2) \log \frac{p(0,1,2)p(1)}{p(0,1)p(1,2)} + p(1,0,2) \log \frac{p(1,0,2)p(0)}{p(1,0)p(0,2)} \\
&+ p(1,1,1) \log \frac{p(1,1,1)p(1)}{p(1,1)p(1,1)} + p(1,1,2) \log \frac{p(1,1,2)p(1)}{p(1,1)p(1,2)}
\end{aligned}
\tag{4}
$$

The joint event patterns of (X_{t+1}, X_t), (X_t, Y_t), and (X_t) overlap depending on (X_{t+1}, X_t, Y_t), which has the most diverse joint event patterns. For instance, in Equation 4, $p(1, 1)$ is repeated two times and $p(1)$ is repeated three times. As FastTENET simultaneously computes the TEs based on the unique pattern count array along the column axis, we need to rearrange the count array to account for patterns that appear multiple times.

As the sub-joint events (X_{t+1}, X_t), (X_t, Y_t), and (X_t) appeared multiple times, the count array of the sub-joint event was repeated to match the same shape as that of the diverse joint event (X_{t+1}, X_t, Y_t). The subpattern array was selected first from the (X_{t+1}, X_t, Y_t) unique pattern array. Then, the unique function was used to create a unique subpattern array and repetition count array from the subpattern array. Each element of the count array from the subpattern array denotes the number of repetitions for each element in the unique pattern count array.

Figure S12 illustrates the example of the unique function to the subpattern array (X_{t+1}, X_t). The repetition count array contains information about the number of repetitions for each pattern; this is necessary to make the shape of the (X_{t+1}, X_t) unique pattern count array matches that of the count array in the joint event (X_{t+1}, X_t, Y_t).

The repetition is conducted with the `repeat` function of NumPy. The repeat function enables the user to specify the number of repetitions for each element of an array (Code 2).

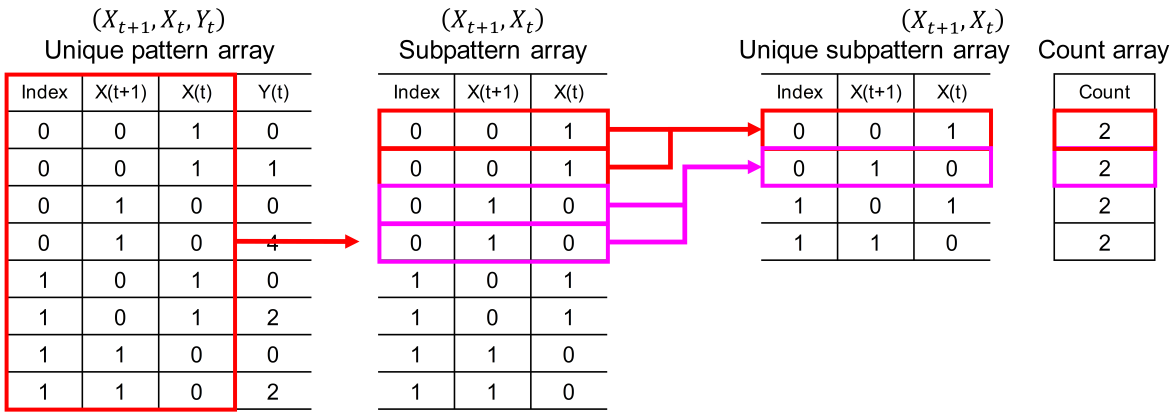

Figure S12. Example of applying the unique function to the subpattern array for creating a repetition count array.

Code 2. Example code of the repeat function in NumPy

```
import numpy as np

count_arr = np.array([0, 1, 2, 3])

repetition_count_arr = np.array([1, 2, 3, 4])

repeated_arr = np.repeat(count_arr, repetition_count_arr)

print(repeated_arr)

# Output:
# array([0 1 1 2 2 2 3 3 3 3])
```

The count arrays of unique patterns can be repeated to fit the case of joint event (X_{t+1}, X_t, Y_t) by utilizing the repeat function (Fig. S13).

In Figure S13, rows of the same color within the pattern are repeated. The repeated count array of the sub-joint event is then matched with the shape of the joint event (X_{t+1}, X_t, Y_t) to enable computations along the column axis. FastTENET utilizes the repeat function of NumPy to repeat the array; therefore each value does not need to have the same number of duplicates. However, repeating a specific value at a specific

(X_{t+1}, X_t, Y_t)

Index	X(t+1)	X(t)	Y(t)	Count
0	0	1	0	2
0	0	1	1	1
0	1	0	0	1
0	1	0	4	1
1	0	1	0	1
1	0	1	2	1
1	1	0	0	2
1	1	0	2	1

(X_{t+1}, X_t)

Index	X(t+1)	X(t)	Count
0	0	1	3
0	0	1	3
0	1	0	2
0	1	0	2
1	0	1	3
1	0	1	3
1	1	0	2
1	1	0	2

(X_t, Y_t)

Index	X(t)	Y(t)	Count
0	0	0	1
0	0	4	1
0	1	0	2
0	1	1	1
1	0	0	1
1	0	2	1
1	1	0	2
1	1	2	1

(X_t)

Index	X(t)	Count
0	0	2
0	0	2
0	1	3
0	1	3
1	0	2
1	0	2
1	1	3
1	1	3

Figure S13. Example of a repeated count array. Identical colors within a pattern indicate that it has been repeated. The colors between patterns are irrelevant.

location is impossible. Therefore, FastTENET also utilizes the `lexsort` and `argsort` functions to align the repeated array to match the order of the unique pattern count array: (X_{t+1}, X_t, Y_t). This process is illustrated in Figure S14 for the case of (X_t) events.

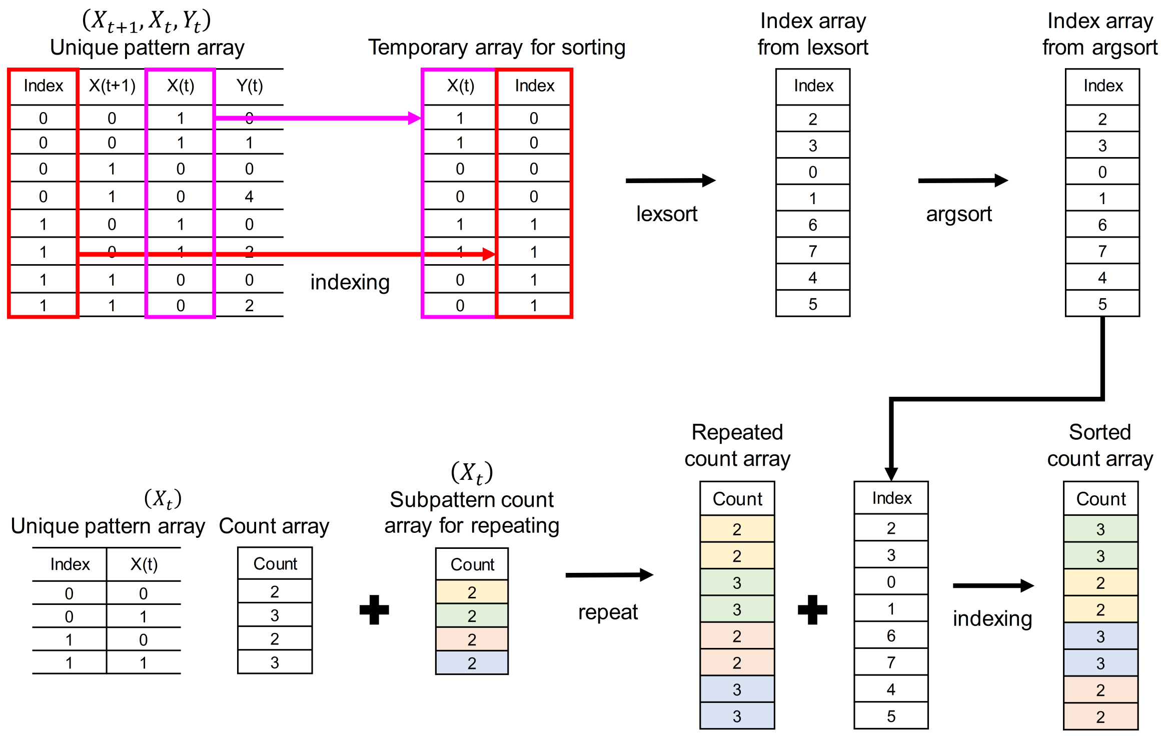

Figure S14. Example of sorting process for the (X_t) event pattern.

In Figure S14, a temporary array (here, the (X_t) event pattern) is indexed for sorting from the (X_{t+1}, X_t, Y_t) unique pattern array. The lexsort function is then applied to the temporary array for sorting it by prioritizing the specified axis and returning the index

of the sorted array. In the example below, the index array from the lexsort function with the (X_t) axis as the primary index axis and the index axis of the unique pattern as the secondary index axis is returned. The argsort function is utilized to obtain an array of indexes that allows sorting according to the unique pattern count array of the diverse joint event. In Figure S14, the repeat function is utilized for the count array and repetition count array. Then, a repeated array is sorted along the index array from the argsort to obtain the sorted count array, which is aligned with the unique pattern count array of the joint event: (X_{t+1}, X_t, Y_t). The final count arrays for computation of the TEs are obtained by the sub-joint events in the same way (Fig. S15).

3.3.4 Computation of transfer entropy

Finally, we divide the count arrays by the total number of observations $t-1$ and calculate each term of the TE along the column axis. Figure S16 shows the process of calculating each term of the TEs. The TEs are computed by adding each term of the TEs along the gene pair index. Each gene pair has a different number of unique patterns, resulting in a different number of joint probabilities required to compute transfer entropy (TE) for each pair. FastTENET utilizes the `bincount` function of NumPy to compute the TE for each gene pair by identifying the gene pair through the gene pair index column (Fig. S17).

(X_{t+1}, X_t, Y_t)

Index	X(t+1)	X(t)	Y(t)	Count
0	0	1	0	2
0	0	1	1	1
0	1	0	0	1
0	1	0	4	1
1	0	1	0	1
1	0	1	2	1
1	1	0	0	2
1	1	0	2	1

(X_{t+1}, X_t)

Index	X(t+1)	X(t)	Count
0	0	1	3
0	0	1	3
0	1	0	2
0	1	0	2
1	0	1	3
1	0	1	3
1	1	0	2
1	1	0	2

(X_t, Y_t)

Index	X(t)	Y(t)	Count
0	1	0	2
0	1	1	1
0	0	0	1
0	0	4	1
1	1	0	1
1	1	2	1
1	0	0	2
1	0	2	1

(X_t)

Index	X(t)	Count
0	1	3
0	1	3
0	0	2
0	0	2
1	1	3
1	1	3
1	0	2
1	0	2

Figure S15. Example of rearranged count arrays.

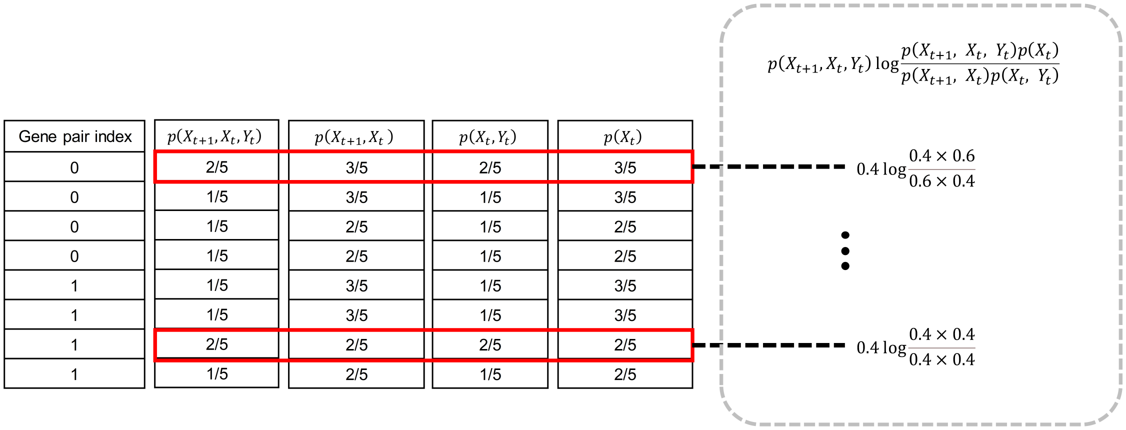

Figure S16. Example of calculating each term of TEs along the column axis.

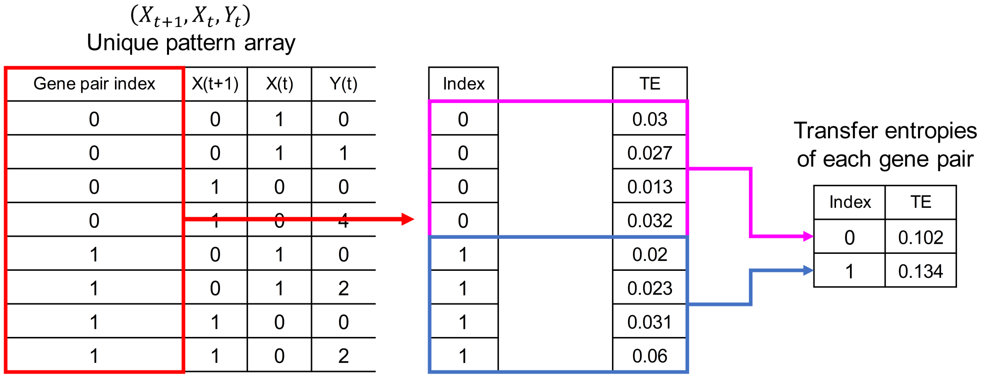

Figure S17. Example of computing TEs using the `bincount` function.

3.4 Parallel processing on manycore processors

Users can utilize FastTENET with its array structure on manycore processors such as GPUs (graphic processing units) or TPUs (tensor processing units). FastTENET utilizes manycore processors by supporting acceleration frameworks such as NumPy, CuPy, Jax, TensorFlow, PyTorch, and PyTorch Lightning. Furthermore, FastTENET offers multiprocessing capabilities from multiple CPUs and manycore processors to accelerate computation. Users can organize batches based on gene pair permutations to perform parallel computations on multiple processors. To accelerate the computation of FastTENET, users can use an appropriate number of processes (CPU cores) and available manycore devices.

3.5 Utilization

3.5.1 Initialization

FastTENET requires the same data preparation as TENET for TE computation. FastTENET requires expression data in CSV format, pseudo-time data, and cell select data in TXT format. Optionally, a transcription factor (TF) file can also be included if needed. When creating and initializing the class, FastTENET accepts file paths to the required data as parameters. The parameters `dpath_exp_data, dpath_trj_data, dpath_branch_data,` and `dpath_tf_data` take the file paths for the expression data, pseudo-time data, cell select data, and TF data respectively. To export the result matrix, the parameter `spath_result_matrix` allows you to enter the file path where the computed transfer entropy array will be saved. FastTENET enables the conversion of expression data in CSV format to a binary file for faster preprocessing when re-utilizing that data. If the `make_binary` parameter is set to True, FastTENET saves a binary file for the expression data.

3.5.2 Execution

After creating a FastTENET class, TE computations can be performed using the `run` function. This function provides parameters for hardware, multiprocessing, discretization method, and more. The `backend` and `device_ids` parameters specify the type and number of hardware to be utilized in the computation. To utilize specific hardware, the `backend` parameter should be set to `"cpu"` or `"gpu"`. When utilizing GPUs or TPUs, users can select a specific manycore acceleration framework such as `"cupy"`, `"jax"`, `"tensorflow"`, `"torch"`, or `"lightning"`. If `"gpu"` is selected, the PyTorch Lightning, which is the default acceleration framework of FastTENET, will be utilized. The `device_ids` parameter should be a list of processor indices or an integer specifying the number of processors to utilize. Entering an integer will automatically allocate the desired number of GPUs, starting with GPU number 0. The parameter `procs_per_device` specifies the number of processes to create per device, with each process assigned to

one CPU core. For example, if `device_ids` is set to 8 and `procs_per_device` is set to 4, FastTENET will utilize 8 manycore processors and 32 CPU cores to compute the TE. If the user utilizes PyTorch Lightning as a framework, this means that the `backend` parameter is set to `"gpu"` or `"lightning"`, the user cannot specify the number of processes by setting `procs_per_device` parameters. Instead, PyTorch Lightning will utilize optimal conditions of CPU resources automatically. If the `backend` parameter is set to `"cpu"`, `procs_per_device` must be set to 1. In this case, the `device_ids` value represents the total number of processes. The `batch_size` specifies the number of TE computations for gene pairs conducted in a single iteration on a single device. Code 3. shows an example of FastTENET execution.

Code 3. Example code for running FastTENET

```
import os.path as osp
import numpy as np
import fasttenet as fte

dpath_exp_data = osp.join(dpath_input, 'exp_data.csv')
dpath_trj_data = osp.join(dpath_input, 'pseudotime_data.txt')
dpath_branch_data = osp.join(dpath_input, 'branch_data.txt')
spath_result_matrix = osp.join(dpath_output, 'result_matrix.txt')

# Create a FastTENET object.
# The file paths for TFs and save are optional.
worker = fte.FastTENET(dpath_exp_data=dpath_exp_data,
                       dpath_trj_data=dpath_trj_data,
                       dpath_branch_data=dpath_branch_data,
                       dpath_tf_data=dpath_tf_data,
                       spath_result_matrix=spath_result_matrix,
                       make_binary=True)

result_matrix = worker.run(backend='gpu',
                           device_ids=8,
                           procs_per_device=4,
                           batch_size=2**15)
```

4 Performance analysis

4.1 Computing systems

Table S1 presents the computing system configurations used for the performance analysis of FastTENET. It provides the system specifications for CPUs and GPUs, including their core counts and memory capacities.

Table S1. Computing system configurations for performance analysis.

System	Device	Product	Specification
System 1	CPU	Intel Xeon Silver 4214R	48 cores<br>512 GB memory
	GPU	NVIDIA RTX 2080Ti	4,352 CUDA cores<br>11 GB memory
System 2	CPU	Intel Core i9-10980XE	36 cores<br>128 GB memory
	GPU	NVIDIA TITAN RTX	4,608 CUDA cores<br>24 GB memory
System 3	CPU	AMD EPYC 7702	128 cores<br>256 GB memory
	GPU	NVIDIA RTX 3090	10,496 CUDA cores<br>24 GB memory
System 4	CPU	Intel Xeon Silver 4214R	48 cores<br>1,024 GB memory
	GPU	NVIDIA RTX A5000	8,192 CUDA cores<br>24 GB memory
System 5	CPU	AMD Ryzen Threadripper Pro 5955WX	32 cores<br>256 GB memory
	GPU	NVIDIA RTX 4090	16,384 CUDA cores<br>24 GB memory
System 6	CPU	Intel Xeon Gold 6338	48 cores<br>400 GB memory
	GPU	NVIDIA A100	6,912 CUDA cores<br>80 GB memory
System 7	CPU	AMD EPYC 7543	32 cores<br>2 TB memory
	GPU	NVIDIA RTX A5000	8,192 CUDA cores<br>24 GB memory
System 8	CPU	AMD EPYC 7542	32 cores<br>1 TB memory
	GPU	NVIDIA RTX A5000	8,192 CUDA cores<br>24 GB memory

4.2 Dataset overview

The mESC and human skin cancer scRNAseq datasets are used to analyze various performance aspects of FastTENET. TENET utilizes pseudo-time as a crucial input, thereby providing a temporal framework that aligns with the dynamic nature of gene expression. The mESC dataset is especially significant as it traces the pseudo-time trajectory from pluripotent stem cells to neural progenitors, whereas the skin cancer datasets describe the progression from normal cells to melanoma. This allows TENET and FastTENET to model and understand the intricate gene interactions during critical transitions in cell state, which is vital for explaining the underlying mechanisms of development and oncogenesis.

The raw dataset of the mESC neural differentiation was downloaded from the GEO database (GEO IDs: GSE107493 (Tuck *et al.*, 2018)). In a study of TENET paper, a dataset consisting of 3,277 highly variable genes from 459 cells was analyzed. Pseudo-time analysis was conducted using the wishbone algorithm (Setty *et al.*, 2016), from a naïve ground state pluripotency (2iL) to differentiation-permissive (SL) to neural precursor cells (NPCs).

The raw datasets of the human skin cancer were downloaded from the GEO database (GEO IDs: GSE144236 (Ji *et al.*, 2020), GSE147944 (Gaydosik *et al.*, 2020), GSE179162 (Godsel *et al.*, 2022), GSE143791 (Kfoury *et al.*, 2021)). The scRNAseq datasets were preprocessed by Scanpy (v.1.9.4). The datasets were integrated utilizing BBKNN (v.1.6.0), and cells from non-skin organs were excluded. The pseudo-time was calculated utilizing PAGA, from normal cells to melanoma. The input data comprised a highly variable set of 37,449 genes, and 20% of the total cells (7,490 cells) were randomly selected.

4.3 Performance metrics

To evaluate the performance of GRN reconstruction algorithms, we define three primary metrics: execution time, discovery score, and AUDS.

1. **Execution time**: Execution time measured in seconds is a basic metric for quantifying the computational speed of an algorithm.

2. **Discovery score**: An important objective of the TENET algorithm is the identification of key players or essential regulators. To evaluate the effectiveness of an algorithm in identifying key regulators, we define the discovery score using the p-value obtained from the hypergeometric test as follows:

$$\text{Discovery score} := -\log_{10}(\text{p-value of hypergeometric test}).$$

The p-value of the hypergeometric test is defined as follows:

$$p\text{-value} = P(X \geq k) = \sum_{i=k}^{\min(K,n)} P(X = i) = \sum_{i=k}^{\min(K,n)} \frac{\binom{K}{i}\binom{N-K}{n-i}}{\binom{N}{n}},$$

where:

- p-value: The probability of observing k or more key regulators in the sample.
- K: The total number of key regulators.
- N: The total number of nodes.
- n: The size of the sample (e.g., the top-k nodes).
- k: The observed number of key regulators in the sample.
- $\binom{a}{b}$: The binomial coefficient, representing the number of ways to choose b key regulators from a nodes.

This formulation allows for a more interpretable metric, where lower p-values correspond to higher discovery scores, indicating a stronger significance in identifying key regulators in GRNs.

3. **AUDS**: AUDS refers to the Area Under the Curve (AUC) of Discovery Scores, which is calculated by increasing the sample size in the hypergeometric test.

4.4 Analysis of computational speed

4.4.1 Analysis of acceleration framework

Computation comparison has been conducted between acceleration frameworks in FastTENET including CuPy, JAX, TensorFlow, PyTorch, and PyTorch Lightning. As we performed various experiments of computation performance of FastTENET, it is observed that the results of execution time on the same datasets, device configurations and parameter conditions are distinct when utilizing different acceleration frameworks. Accordingly, we conducted the benchmark experiment for each framework by measuring the execution time of each function of the FastTENET algorithm.. The algorithm of FastTENET can be grouped into four principal parts: unique function ('**unique**'), repeat function ('**repeat**'), sort function ('**sort**'), and other functions ('**etc**'). We combine the 'sort' and 'etc' parts as those have less execution time than the others. In the experiment, the execution time of the 'unique', 'repeat', and 'etc' parts was measured for each framework. We have utilized System 4 in Table S1 which has NVIDIA RTX A5000 GPU devices. In the experiment, we have utilized single GPU device and the parameters were set to a batch size of 65,536 and a single process per device for the mESC dataset, and a batch size of 4,096 and a single process per device for the skin cancer dataset.

Figure S18 shows the execution time for each framework. The radius of the pie chart is proportional to the execution time, thus indicating that the larger the pie chart, the longer the execution time when utilizing the respective framework. Additionally, the figure shows that the proportion of execution time of each part in each framework. Figure S18 shows that the best framework on computation performance during those frameworks is PyTorch Ligntning in both datasets. CuPy framework exhibited the longest execution time of all frameworks on the mESC dataset. Further-

more, unlike other frameworks, the part that takes the largest percentage of execution time is ‘repeat’. Otherwise, the part that the largest percentage of execution time is ‘unique’ in TensorFlow, PyTorch, PyTorch Lightning frameworks. JAX framework also exhibits ‘unique’ part as the largest percentage of execution time, but more balanced distributed percentages are observed. These result indicates the difference of implementation of parts in each framework occurs different computation performance regardless of FastTENET algorithm. Table S2 shows the detail execution time of the experiments.

Table S2. Comparison of execution times for computational parts across manycore acceleration frameworks in FastTENET.

**Framework**	**Dataset**					
	mESC neural differentiation			Skin cancer		
	**Part**	**Execution time**	**Proportion**	**Part**	**Execution time**	**Proportion**
CuPy	unique	117.9 sec.	1.4%	unique	35.4 sec.	10.1%
	repeat	8,251.2 sec.	96.6%	repeat	312.6 sec.	88.8%
	etc	173.1 sec.	2.0%	etc	3.8 sec.	1.1%
	**Total**	**8,542.2 sec.**	100.0%	**Total**	**351.9 sec.**	100.0%
JAX	unique	3,003.5 sec.	59.7%	unique	802.9 sec.	61.3%
	repeat	574.4 sec.	11.4%	repeat	159.0 sec.	12.1%
	etc	1,453.4 sec.	28.9%	etc	348.8 sec.	26.6%
	**Total**	**5,031.3 sec.**	100.0%	**Total**	**1,310.7 sec.**	100.0%
TensorFlow	unique	679.4 sec.	88.7%	unique	205.6 sec.	89.7%
	repeat	10.3 sec.	1.4%	repeat	1.9 sec.	0.8%
	etc	76.1 sec.	9.9%	etc	21.6 sec.	9.5%
	**Total**	**765.8 sec.**	100.0%	**Total**	**229.1 sec.**	100.0%
PyTorch	unique	70.5 sec.	96.6%	unique	30.9 sec.	97.6%
	repeat	1.4 sec.	1.9%	repeat	0.1 sec.	0.3%
	etc	1.1 sec.	1.5%	etc	0.6 sec.	2.1%
	**Total**	**73.0 sec.**	100.0%	**Total**	**31.6 sec.**	100.0%
PyTorch Lightning	unique	35.8 sec.	89.7%	unique	13.9 sec.	98.2%
	repeat	0.9 sec.	0.8%	repeat	0.1 sec.	0.4%
	etc	0.6 sec.	9.5%	etc	0.1 sec.	1.4%
	**Total**	**37.3 sec.**	100.0%	**Total**	**14.2 sec.**	100.0%

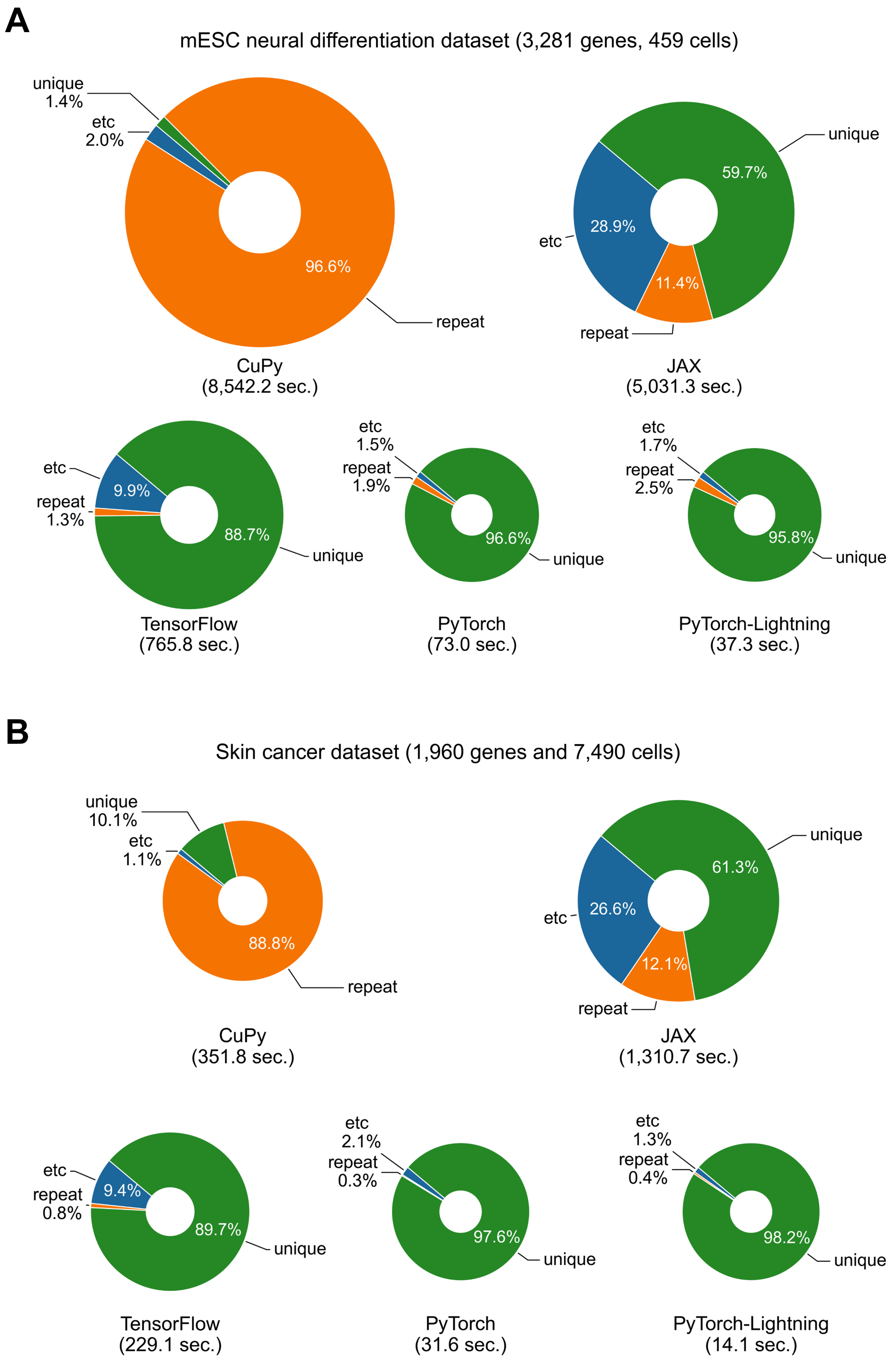

Figure S18. Comparison of execution times among manycore acceleration frameworks of FastTENET on the **(A)** mESC neural differentiation and **(B)** skin cancer datasets.

4.4.2 Impact of the number of processors

We performed additional computation comparison experiments by utilizing systems having the diverse GPU and CPU configurations listed in Table S1. A comparison experiment was conducted under two distinct conditions. The experiment involved the utilization of a single GPU and a variable number of processes per GPU (Fig. S19). The number of processes per GPU device was set to 1, 2, 4, 8, 16, and 32. The batch size of each process was determined by the given dataset to maximize GPU memory usage. Because each system has a distinct GPU memory size, the batch size of each system is accordingly variable. The experiments were repeated 10 times for both datasets. The relative performance of FastTENET was obtained by dividing its execution time by that of the TENET under the multiprocessing condition of 32 CPU cores. In experiment, PyTorch is utilized as a acceleration framework.

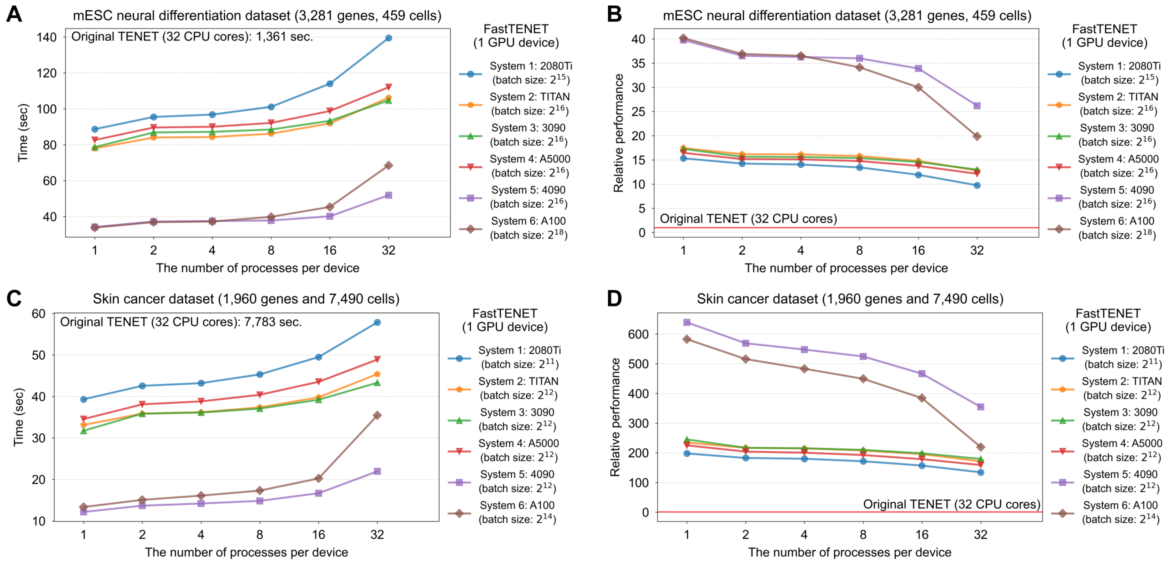

Figure S19. Impact of number of processes per GPU device on computational speed. **(A)** Execution time and **(B)** relative performance of FastTENET on the mESC dataset. **(C)** Execution time and **(D)** relative performance of FastTENET on the skin cancer dataset. The configurations of each system are detailed in Table S1. All experiments of TENET were conducted with Intel Xeon Silver 4214R 32 CPU cores.

Figure S19 shows that the performance is decreased when the number of GPUs exceeded a certain threshold. This result indicates that an optimal condition exists for the number of GPUs and processes per GPU device along the dataset, regardless of the

system configurations.

4.4.3 Impact of batch size

We performed a computation comparison to investigate the impact of different batch sizes on the performance of FastTENET with the mESC and skin cancer datasets. We utilize two systems that have different GPUs including NVIDIA RTX A5000 and RTX 2080Ti. Furthermore, both systems have the same CPU and memory configurations. In the mESC dataset, as the memory size of the GPU device of each system is distinct, we set batch size conditions from 512 to 65,536 for RTX A5000 and from 256 to 32,768 for RTX 2080Ti. In the case of the skin cancer dataset, the batch size condition was set to range from 32 to 4,096 for RTX A5000, and from 16 to 2,048 for RTX 2080Ti. The number of GPU devices was set to the same conditions in both systems: 1, 2, 4, and 8. The experiments were repeated five times for the mESC dataset and ten times for the skin cancer dataset. The accelerating framework of FastTENET is set to PyTorch Lightning in both GPU systems. Figure S20A and S20B show the execution time of FastTENET on RTX A5000 GPU and RTX 2080Ti GPU on the mESC dataset, respectively. Both subplots show that execution times are decreased when batch sizes are increased. In the case of utilizing 8 GPU devices, the performance gains resulting from an increase in batch size are less dramatic than those observed with a single GPU device. Figure S20C and S20D show the execution time of FastTENET on the skin cancer dataset. The same pattern of execution time graphs is observed in both subplots. This result indicates that utilizing the maximum memory of GPU devices leads to an increase in performance in FastTENET.

4.4.4 Impact of data size

We have conducted additional computation comparison experiments with different sizes of data matrix conditions by creating synthetic expression datasets. To create a synthetic dataset, we generate a synthetic graph by utilizing `barabasi_albert_graph` func-

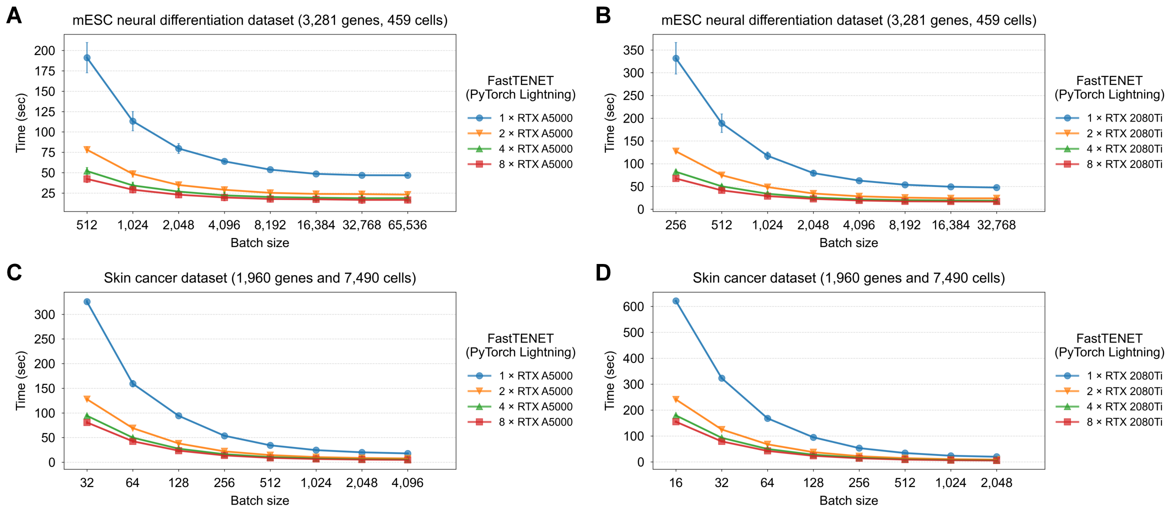

Figure S20. Impact of batch size on computational speed. Execution times of FastTENET on the mESC dataset using **(A)** NVIDIA RTX A5000 and **(B)** NVIDIA RTX 2080Ti. Execution times of FastTENET on the skin cancer dataset using **(C)** NVIDIA RTX A5000 and **(D)** NVIDIA RTX 2080Ti.

tion of NetworkX Python package. Figure S21A shows the example graph generated using Barabási-Albert preferential attachment algorithm (`barabasi_albert_graph` function of NetworkX). After generating the graph, we select the main node which has the most links in nodes of the graph and create the expression pattern of the main node (Fig. S21B). Then, we create synthetic expression data by simulating expression flow with difference equation in Equation 5.

$$X(t+1) = \alpha W X(t) + (1-\alpha) B \tag{5}$$

where $X(t)$ denotes expression values of nodes at time t and W is the weight matrix between nodes which denotes the link of the graph. B is the basal activity of each node. We created W matrix from a synthetic graph by utilizing `to_numpy_array` function of NetworkX and we set the weights of links to 1. The α is a parameter to control the influence of the current state and basal activity on the next state, which is set to 0.8.

In this analysis, we create five synthetic expression datasets with varying the number of node n (i.e., the number of genes) and the length of time t (i.e., the number of cells). The sizes of the synthetic data matrices are defined as 128×512, 256×1,024, 512×2,048,

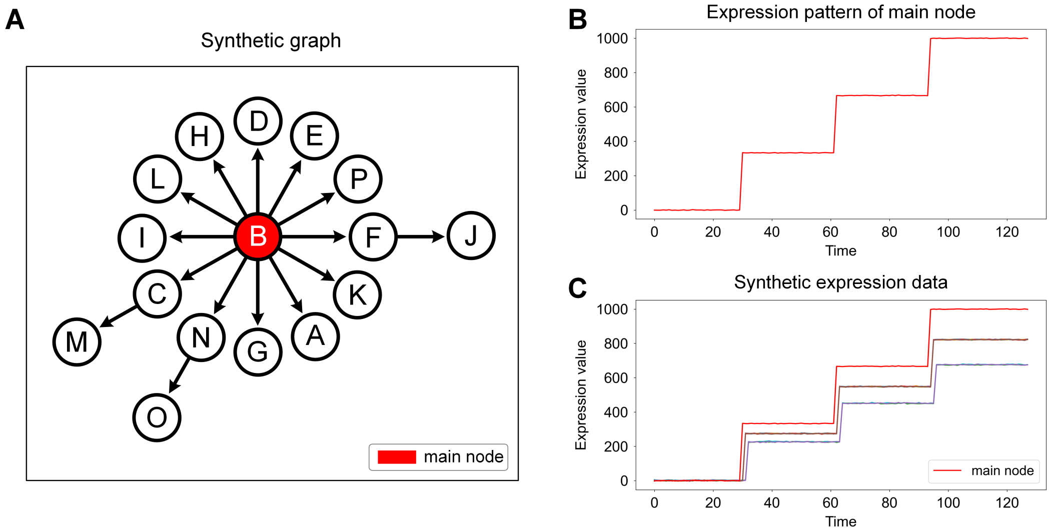

Figure S21. An example of synthetic dataset. **(A)** A synthetic graph generated using Barabási-Albert preferential attachment algorithm. **(B-C)** A synthetic expression data of (B) main node and (C) the other nodes in the synthetic graph.

1,024×4,096, and 2,048×8,192. The graphs of each synthetic dataset have been created using the aforementioned synthetic dataset creation method. We have created same expression pattern of main node and scaled it along with time t of each dataset. The experiences are conducted by utilizing NVIDIA RTX A5000 from System 4 in Table S1. We set the processes per device, number of GPU devices, batch size, and acceleration framework parameters of FastTENET to 1, 2, 4, 8, 16, and 32, 1-8, 2^7-2^{14}, and PyTorch, respectively.

Figure S22A shows the execution time with different data matrices on processes per device conditions. As the number of processes per device increases, the computation performance decreases across all datasets. This indicates that adding more processes may introduce overhead, leading to increased execution time.

In Figure S22B, for the datasets which have a data matrix, 512×2,048, 1,024×4,096, and 2,048×8,192, the computation performance increases steadily as the number of GPU devices increases. Otherwise, for the datasets that have a data matrix, 128×512, and 256×1,024, the performance decreases slightly as the number of GPU devices increases. This indicates that the acceleration of computation performance by parallel computing impact more to bigger size of datasets than smaller size of datasets. Fig-

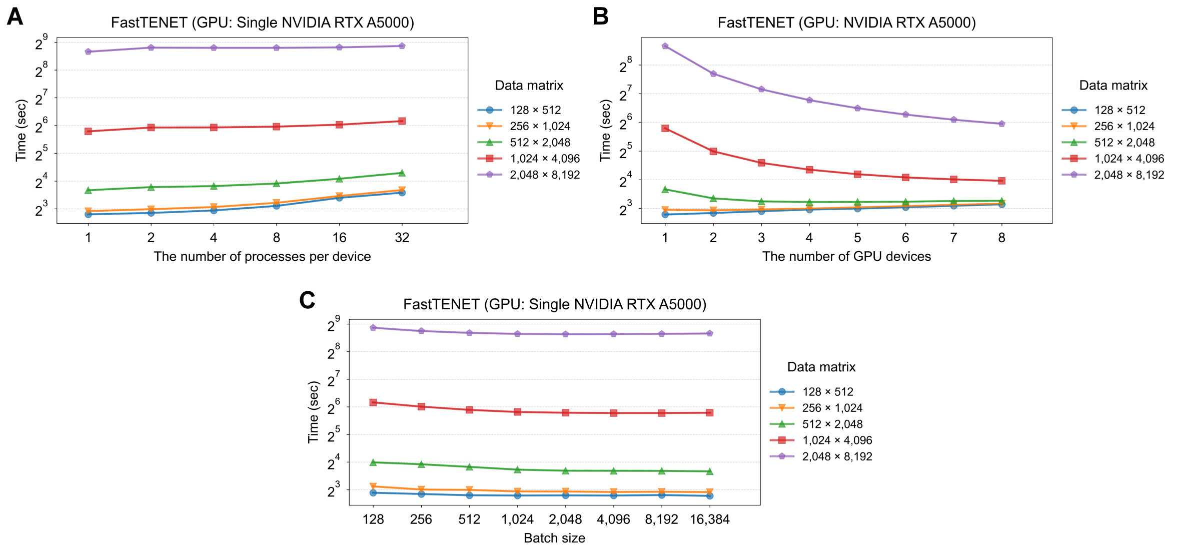

Figure S22. Execution times of FastTENET for different sizes of data matrices with respect to **(A)** the number of processes per device parameter, **(B)** the number of GPU device parameter, and **(C)** the batch size.

ure S22C shows the impact of execution time with different data matrices on various batch sizes. The computation performance decreases across all datasets when the batch size increases, but the impact of acceleration is less than other results.

4.4.5 Impact of expression patterns

We performed additional computation comparison experiments by creating synthetic expression datasets with different expression patterns. The method of creating synthetic datasets is identical to that described in section 4.4.4. In this experiment, we have created four synthetic expression datasets with the same size of the data matrix, 3,000 nodes×8,192 times. We have generated four distinct expression patterns for the main node to create varying numbers of unique patterns in the synthetic datasets (Fig. S23A). The computation of transfer entropies on the synthetic datasets was performed using a single NVIDIA RTX A5000 GPU device. We set the batch size, and acceleration framework parameters to 32,768, and PyTorch Lightning, respectively. In addition, we have measured the execution time of the 'unique', 'repeat', and 'etc' parts in each framework as same as section 4.4.1.

Figure S23B shows the execution time for each synthetic dataset. The synthetic

dataset of Pattern 4, which exhibited the longest execution time (Figure S23B), has the largest number of unique patterns among the four datasets (Table S3). The execution time of the `unique` function part in each dataset constitutes the largest proportion of the total execution time across all synthetic datasets. These results indicate that an increased number of unique patterns in the expression dataset can lead to a decrease in the computational performance of FastTENET, even when the size of the data matrix remains unchanged.

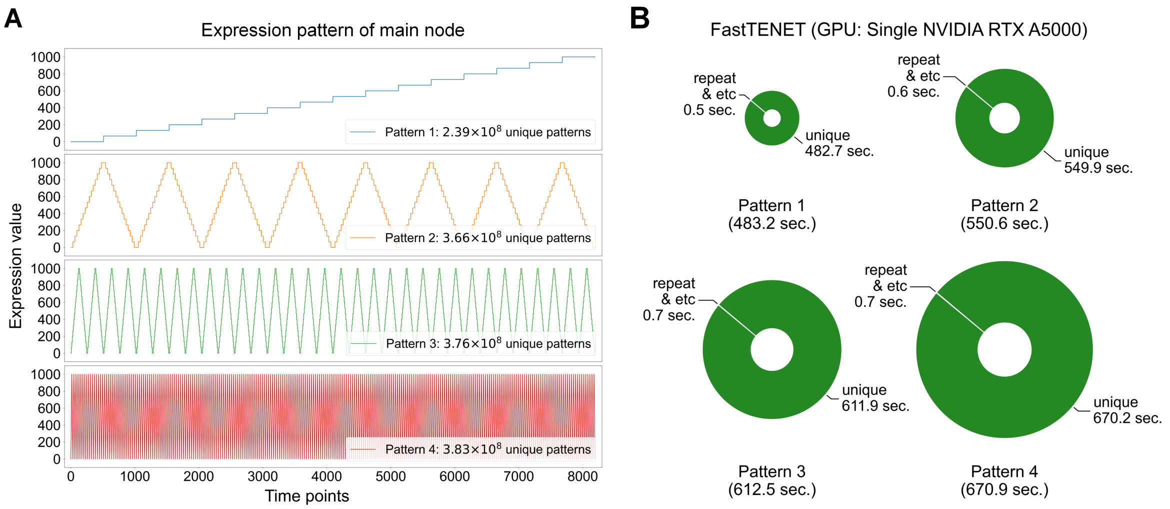

Figure S23. **(A)** Four distinct patterns of expression dynamics for the main node in the graph presented in Figure S21. **(B)** Execution times of FastTENET for these four different expression patterns.

Table S3. The number of unique patterns associated with the four joint events in the synthetic datasets of Figure S23.

Joint event	Synthetic dataset			
	Pattern 1	Pattern 2	Pattern 3	Pattern 4
(X_t)	36,002,995	36,005,994	36,071,972	36,297,930
(X_t, Y_t)	58,317,086	91,375,172	94,906,662	95,249,458
(X_{t+1}, X_t)	63,245,911	93,070,966	93,169,933	94,403,855
(X_{t+1}, X_t, Y_t)	81,340,799	145,355,901	151,579,889	157,344,270
**Total**	238,906,791	365,808,033	375,728,456	383,295,513

Interestingly, the execution time for the skin cancer dataset is much lower than that for the mESC neural differentiation dataset (Figure S24), even though the size of the

skin cancer dataset is approximately 9.7 times larger than that of the mESC dataset (i.e., 1,960×7,490 = 14,680,400 vs. 3,281×459 = 1,505,979). As demonstrated with the synthetic datasets in Figure S23) and Table S3, the execution time of FastTENET depends on the number of unique patterns associated with the joint events identified from gene expression data. Therefore, we have also analyzed the number of unique patterns in the two scRNAseq datasets. Table S4 shows that the number of unique patterns associated with the joint events in the skin cancer is much less than those of the mESC dataset. These results suggest that the number of unique patterns associated with the joint events, rather than the data size, determines the execution time of FastTENET.

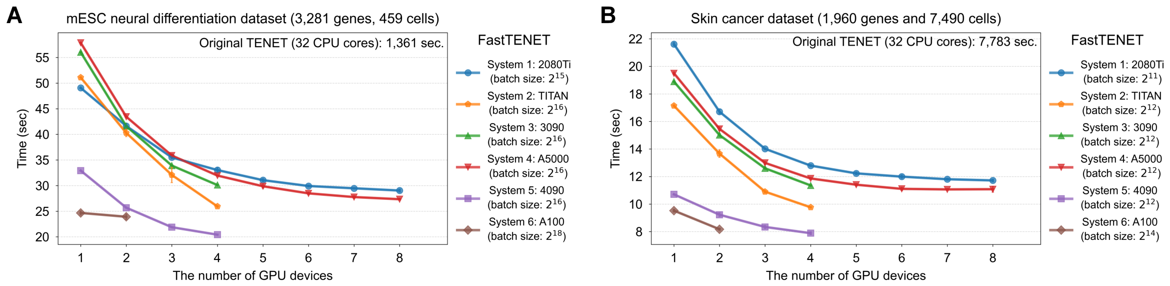

Figure S24. **(A)** Execution times of FastTENET on the mESC dataset and **(B)** Skin cancer dataset. The Intel Xeon Silver 4214R (2.4 GHz) CPU was used for all experiments of the original TENET. All experiments of TENET were conducted with 32 CPU cores.

Table S4. The number of unique patterns associated with the joint events in the mESC neural differentiation and skin cancer datasets.

**Joint event**	**Dataset**	
	mESC neural differentiation	Skin cancer
(X_t)	86,355,840	2,497,251
(X_t, Y_t)	181,186,736	7,432,878
(X_{t+1}, X_t)	189,151,040	8,321,163
(X_{t+1}, X_t, Y_t)	298,307,128	18,255,544
**Total**	755,000,744	36,506,836

4.4.6 Evaluation of GRN algorithms for computational speed

To evaluate FastTENET performance, we benchmarked GRN inference algorithms GENIE3 (Huynh-Thu *et al.*, 2010), GRNBoost2 (Moerman *et al.*, 2019), GRISLI (Aubin-Frankowski and Vert, 2020), SCODE (Matsumoto *et al.*, 2017), LEAP (Specht and Li, 2017), SCRIBE (Qiu *et al.*, 2020) using BEELINE (Pratapa *et al.*, 2020) and TENET (Kim *et al.*, 2021). GENIE3 predicts gene interactions using tree-based ensemble regression, while GRNBoost2 enhances scalability through gradient boosting machine (GBM) regression for large scRNAseq datasets. Many algorithms adopt a time-involved approach to improve the accuracy of GRN reconstruction. GRISLI and SCODE employ ordinary differential equation models with pseudo-time to estimate gene expression dynamics. LEAP utilizes pseudo-time information with lagged correlations, while SCRIBE and TENET leverage pseudo-time information to apply transfer entropy for inferring causal relationships. These GRN reconstruction algorithms, commonly used in the single-cell field, were specifically selected because they require only gene expression data and pseudo-time information. In contrast, other GRN algorithms were excluded because they require additional input data or training sets.

We conducted benchmarking experiments using synthetic datasets of varying sizes (genes × cells) and compared the results of eight GRN reconstruction algorithms. While all other algorithms were operated on CPUs, FastTENET was accelerated using a GPU device. The synthetic dataset benchmark was executed on System 7, with CPU cores limited to 8 and a single GPU allocated for FastTENET (Table S1).

Figure S25 shows the execution times of GRN reconstruction algorithms on the synthetic datasets. As the data size increased, the computational advantage of FastTENET became increasingly pronounced. FastTENET completed the task in just a few minutes for the largest dataset (2,048 genes×8,192 cells), whereas all other algorithms required several hours, with times reaching up to 46 hours, and some algorithms were unable to complete the calculations within 48 hours. Notably, TENET demonstrated fast performance for small datasets, but its performance dropped dramatically

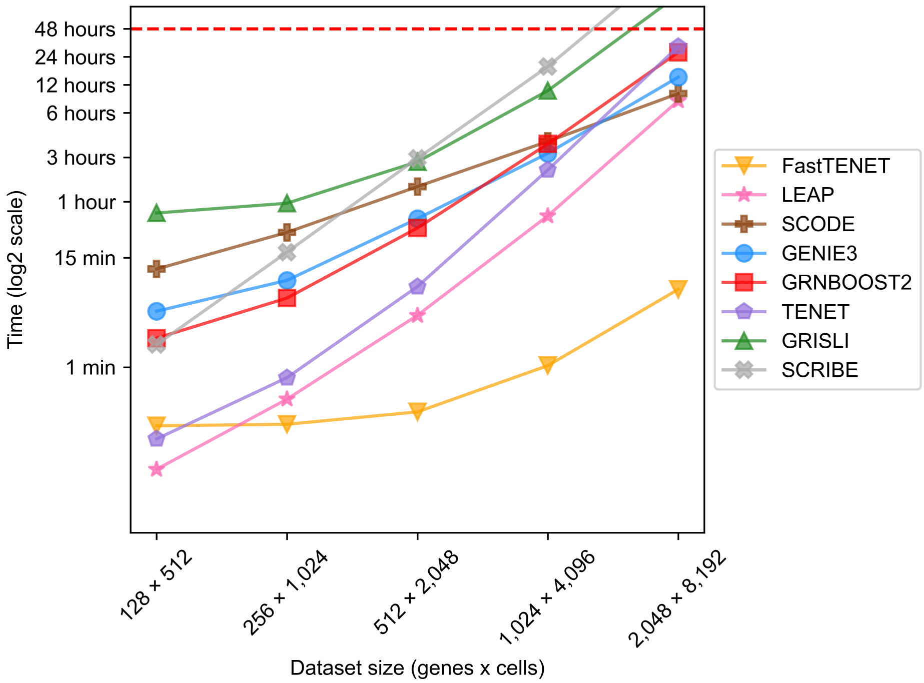

Figure S25. Execution times of GRN reconstruction algorithms on synthetic datasets.

as the data size increased. This limitation in TENET underscores the necessity for FastTENET. FastTENET consistently outperformed all other algorithms, exhibiting speed improvements of several times, particularly on larger datasets.

4.5 Approximation of transfer entropy

4.5.1 Comparison of approximation results

The TE approximation results obtained from FastTENET and TENET may differ, as both algorithms employ distinct discretization methods for approximating TE values. To compare the approximation results, GRNs were reconstructed from each tool and the outdegree for each gene was subsequently computed. The top 20 genes were ranked based on their outdegree, and the correlation between the outdegree values from FastTENET and TENET was subsequently analyzed. Figure S26 shows the results of the comparison experiments between FastTENET and TENET.

As shown in Figure S26A and S26B, the top 20 genes predicted by FastTENET are highly consistent with those predicted by TENET. Specifically, FastTENET accurately

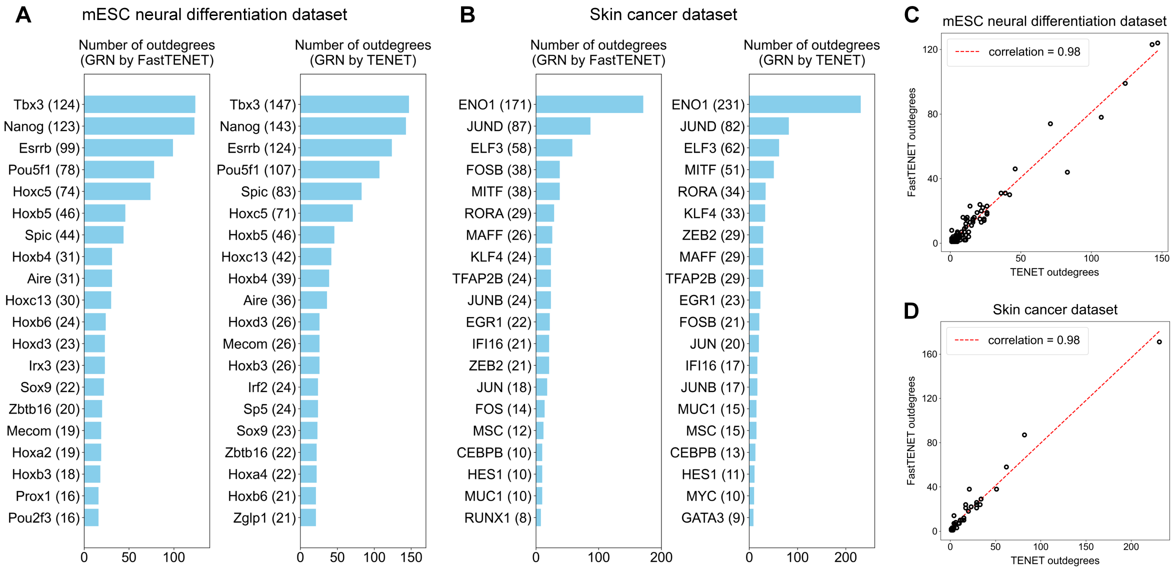

Figure S26. Comparison between FastTENET and the original TENET. The top outdegree lists of the GRN generated by FastTENET and TENET are compared for **(A)** the mESC dataset and **(B)** the skin cancer dataset. Additionally, the correlations of outdegrees between FastTENET and TENET are presented for **(C)** the mESC dataset and **(D)** the skin cancer dataset.

identifies key genes ranked highly by TENET, such as 'Tbx3', 'Nanog', 'Esrrb', and 'Pou5f1' in the mESC dataset, and 'ENO1', 'JUND', and 'ELF3' in the skin cancer dataset. The correlation between the outdegree values from FastTENET and TENET was computed for both datasets, resulting in a correlation coefficient of 0.98 in each case, indicating a strong positive linear relationship between the predictions of FastTENET and TENET (Figs S26C and S26D).

4.5.2 Comparison of smoothing and discretizing functions

Regardless of computational speed, the most significant difference between the original TENET and FastTENET lies in their smoothing and discretizing methods. Although FastTENET operates much faster than the original TENET, it must not sacrifice one of the important advantages of the original TENET: the ability to identify key regulators (or master regulators) in GRNs. Therefore, we have analyzed various combinations of discretization and smoothing functions to evaluate the performance of FastTENET in identifying hub regulators, in comparison to TENET.

To evaluate the impact of smoothing and discretizing functions on discovering the key regulators in FastTENET, we have implemented four discretizing functions and two smoothing functions. In signal processing, a smoothing function is a method to reduce the variability of data or signals, resulting in a smoother representation. Smoothing is utilized to eliminate noise and to clarify the overall patterns within the signal. Due to the inherent noise in gene expression data, smoothing methods are useful for enhancing data quality. We have implemented two smoothing functions, namely 'Moving Average' and 'Exponential Weight'. These functions are applied to the discretized expression data. The following provides detailed information on the smoothing functions implemented in FastTENET.

- **Moving Average**: This method calculates the average of a fixed number of data points by sliding the window and replacing the central data point with this average.
- **Exponential Weight**: This method increases responsiveness to recent changes in the data by assigning greater weight to more recent data points.

The following provides detailed information on the discretizing functions implemented in FastTENET.

- **FSBW-L**: Fixed-size bins are determined based on the standard deviation of expression values for each gene. The bins are then shifted to the left by subtracting half of the standard deviation.
- **FSBN**: Fixed-size bins are linearly spaced based on the specified number of bins. The number of bins for each gene is calculated by dividing the range of expression values by the standard deviation.
- **FSBQ**: Fixed-size bins are determined according to the specified quantiles. The number of quantiles for each gene is calculated by dividing the range of expression values by the standard deviation.
- **K-means**: Expression values are discretized using k-means clustering. The number of clusters for each gene is determined by dividing the range of expression values by the standard deviation.

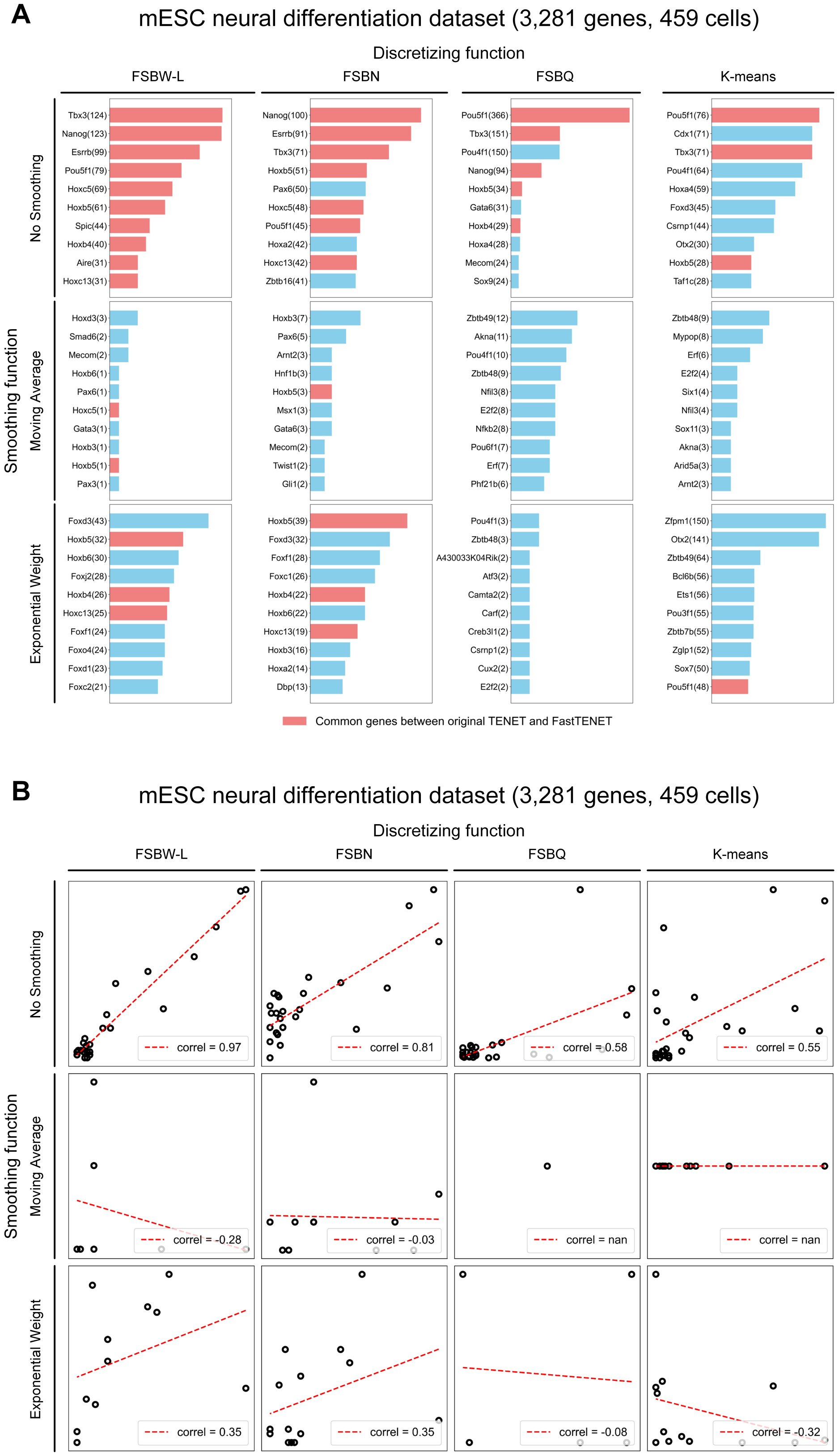

Figure S27. Impact of smoothing and discretizing functions on discovering hub regulators from the mESC neural differentiation dataset. **(A)** The top 10 genes ordered by outdegree. **(B)** Correlation of outdegrees.

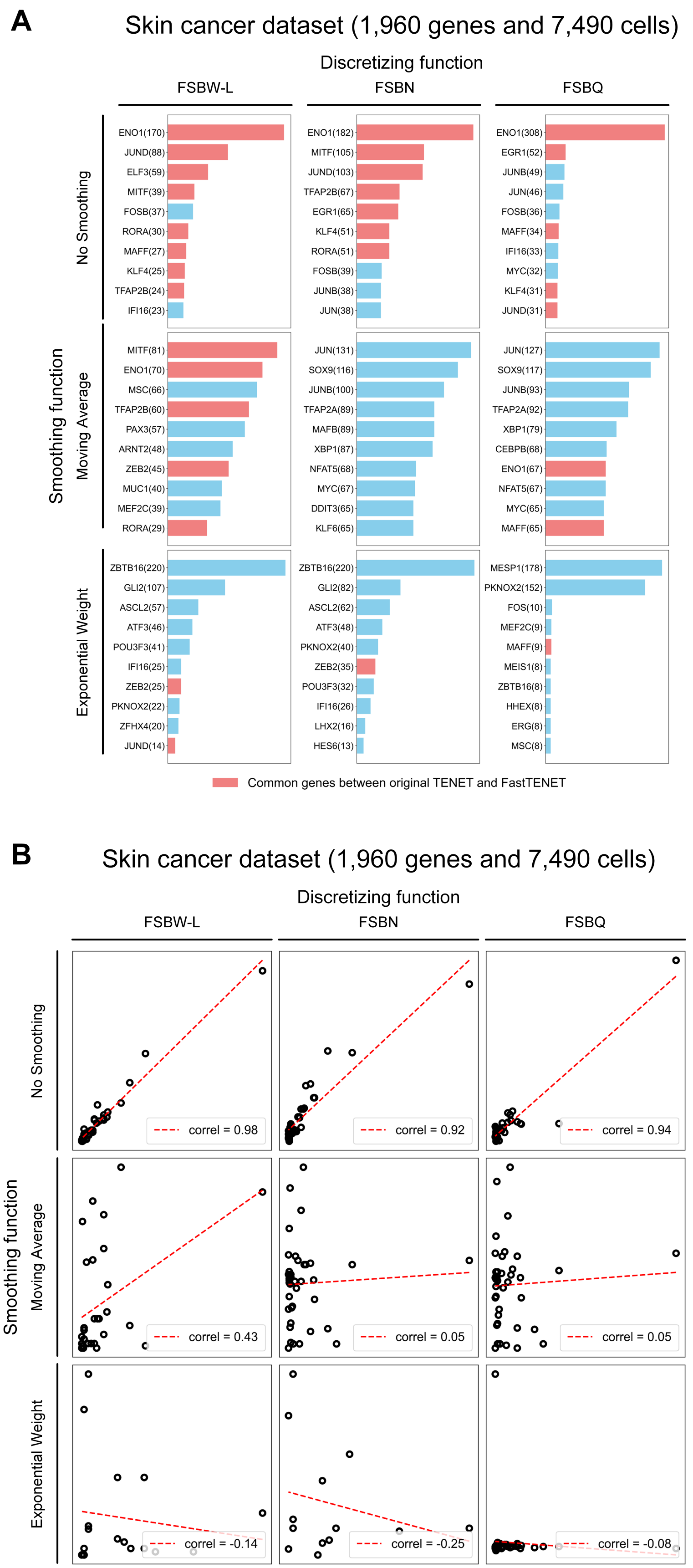

Figure S28. Impact of smoothing and discretizing functions on discovering hub regulators from the skin cancer dataset. **(A)** The top 10 genes ordered by outdegree. **(B)** Correlation of outdegrees.

Figures S27A and S28A show the top 10 hub genes found by FastTENET from the mESC and skin cancer datasets, respectively. The common genes between the original TENET and FastTENET are highlighted in red. These results indicate that two smoothing functions negatively impact the similarity between the original TENET and FastTENET, while FSBW-L discretizing function brings FastTENET closer to the original TENET. Additionally, the correlations of outdegrees between the original TENET and FastTENET support these findings, with the highest correlation observed when using the FSBW-L discretizing function without smoothing (Figs S27B and S28B).

4.5.3 Evaluation of GRN algorithms for transcription factor discovery

To evaluate the GRN reconstruction algorithms, we assessed their ability to identify key transcription factors from the mESC dataset by determining whether the top-k hub nodes (i.e., genes) suggested by each algorithm were present in a reference set of known TFs, using the discovery score as a metric. The skin cancer dataset was excluded from this experiment due to the lack of a reference set for TFs specific to skin cancer. This benchmarking experiment was conducted on System 8 with CPU cores limited to 8, and a single GPU allocated for FastTENET(Table S1).

FastTENET and TENET consistently achieved high discovery scores across the top-k gene set (Figs S29A and S29B). Additionally, we calculated the AUDS to quantify the discovery of TFs, increasing the sample size (i.e., the top-k nodes suggested by algorithm). FastTENET outperformed other algorithms, demonstrating both superior performance in discovery of key regulators and a notable advantage in computational speed (Figs S29C and S29D). For example, in the validation of the top 5 key regulators, FastTENET, TENET, SCRIBE, SCODE, and GRNBOOST2 demonstrated similarly high AUDS. However, FastTENET was up to 20,000 times faster than the other algorithms (Fig. S29D). Furthermore, by employing various combinations of discretizing and smoothing functions, FastTENET using FSBN without smoothing achieved the highest AUDS (i.e., 16.2) from the mESC dataset (Fig. S30).

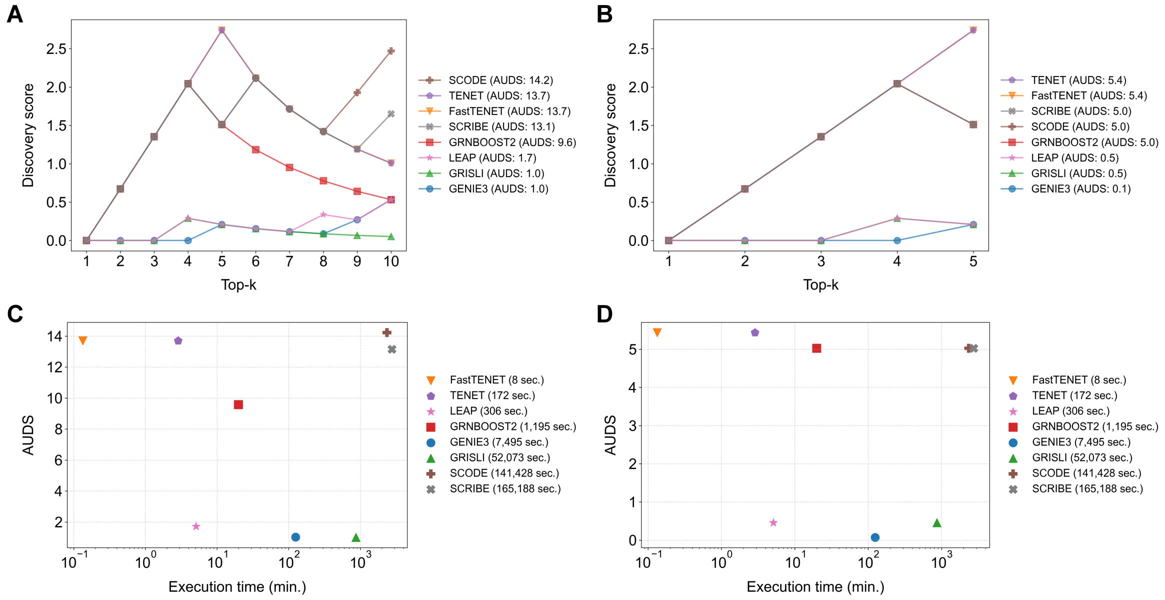

Figure S29. Comparison of GRN reconstruction algorithms for identifying transcription factors from the mESC dataset, based on discovery score and AUDS. **(A-B)** Discovery score evaluated for (A) the top 10 and (B) the top 5 nodes suggested by each GRN algorithm. **(C-D)** AUDS versus execution time for (C) the top 10 and (D) the top 5 nodes suggested by each GRN algorithm.

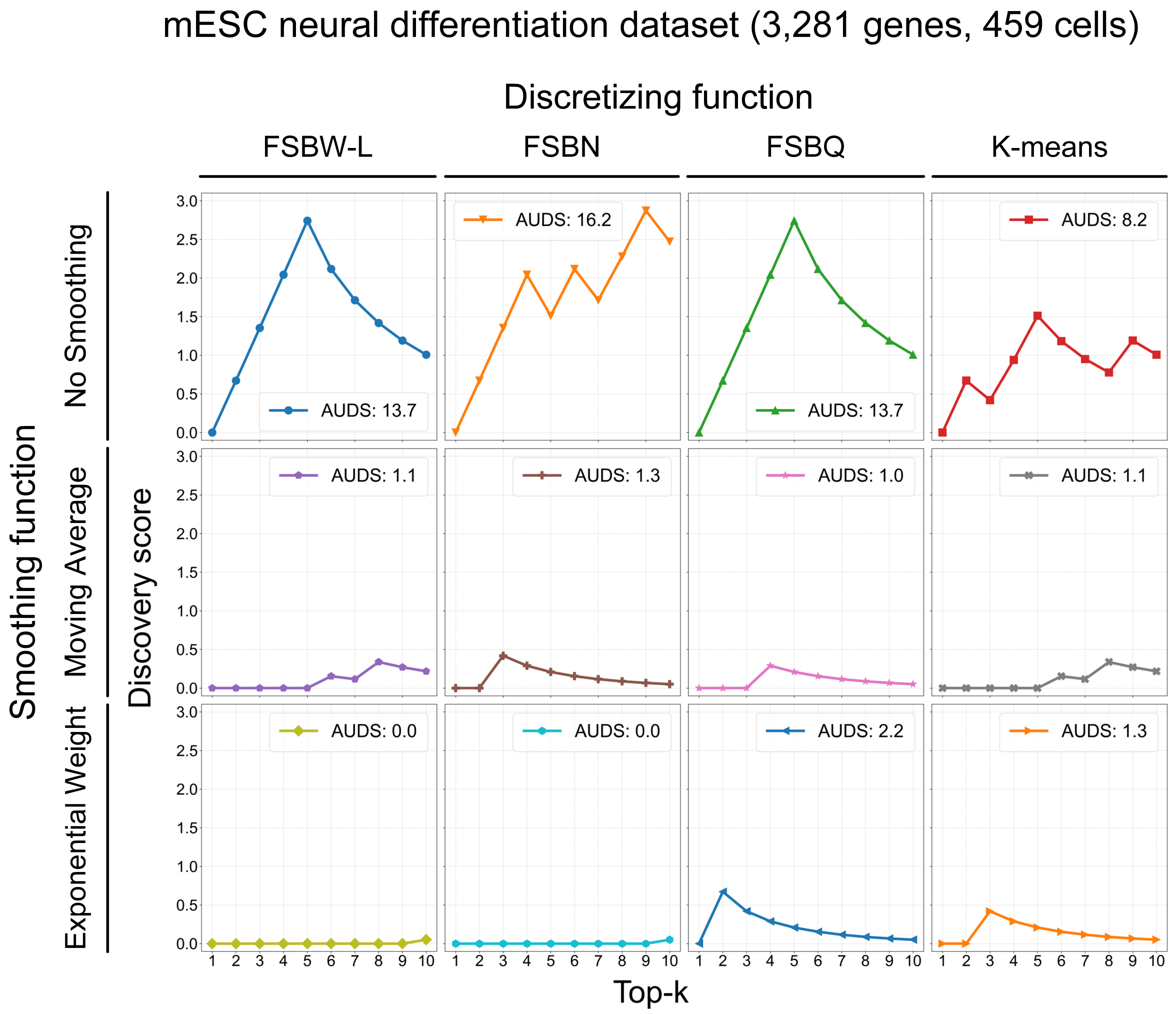

Figure S30. Impact of discretizing and smoothing functions on the discovery score achieved by FastTENET.

References

Aubin-Frankowski, P.-C. and Vert, J.-P. (2020). Gene regulation inference from single-cell RNA-seq data with linear differential equations and velocity inference. *Bioinformatics*, **36**(18), 4774–4780.

Gaydosik, A. M. *et al.* (2020). Genome-wide transcriptome analysis of the STAT6-regulated genes in advanced-stage cutaneous T-cell lymphoma. *Blood, The Journal of the American Society of Hematology*, **136**(15), 1748–1759.

Godsel, L. M. *et al.* (2022). Translational implications of Th17-skewed inflammation due to genetic deficiency of a cadherin stress sensor. *The Journal of Clinical Investigation*, **132**(3).

Huynh-Thu, V. A. *et al.* (2010). Inferring regulatory networks from expression data using tree-based methods. *PloS One*, **5**(9), e12776.

Ji, A. L. *et al.* (2020). Multimodal analysis of composition and spatial architecture in human squamous cell carcinoma. *Cell*, **182**(2), 497–514.

Kfoury, Y. *et al.* (2021). Human prostate cancer bone metastases have an actionable immunosuppressive microenvironment. *Cancer Cell*, **39**(11), 1464–1478.

Kim, J. *et al.* (2021). TENET: gene network reconstruction using transfer entropy reveals key regulatory factors from single cell transcriptomic data. *Nucleic Acids Research*, **49**(1), e1.

Matsumoto, H. *et al.* (2017). SCODE: an efficient regulatory network inference algorithm from single-cell RNA-seq during differentiation. *Bioinformatics*, **33**(15), 2314–2321.

Moerman, T. *et al.* (2019). GRNBoost2 and Arboreto: efficient and scalable inference of gene regulatory networks. *Bioinformatics*, **35**(12), 2159–2161.

Pratapa, A. *et al.* (2020). Benchmarking algorithms for gene regulatory network inference from single-cell transcriptomic data. *Nature Methods*, **17**(2), 147–154.

Qiu, X. *et al.* (2020). Inferring causal gene regulatory networks from coupled single-cell expression dynamics using scribe. *Cell Systems*, **10**(3), 265–274.

Setty, M. *et al.* (2016). Wishbone identifies bifurcating developmental trajectories from single-cell data. *Nature Biotechnology*, **34**(6), 637–645.

Specht, A. T. and Li, J. (2017). LEAP: constructing gene co-expression networks for single-cell RNA-sequencing data using pseudotime ordering. *Bioinformatics*, **33**(5), 764–766.

Tuck, A. C. *et al.* (2018). Distinctive features of lincRNA gene expression suggest widespread RNA-independent functions. *Life Science Alliance*, **1**(4).
